# Metabolomic profiles and health-promoting potential of *Euchresta japonica* tissues revealed by widely targeted metabolomics

**DOI:** 10.3389/fpls.2025.1537273

**Published:** 2025-05-01

**Authors:** Liai Xu, Xi Liu, Xiangdong Pan, Sinan Xu, Qinglian Wu, Chengyi Ma, Zupei Lei, Yeqing Ying

**Affiliations:** ^1^ Key Laboratory of Quality and Safety Control for Subtropical Fruit and Vegetable, Ministry of Agriculture and Rural Affairs, Collaborative Innovation Center for Efficient and Green Production of Agriculture in Mountainous Areas of Zhejiang Province, College of Horticulture Science, Zhejiang A&F University, Hangzhou, Zhejiang, China; ^2^ Wuyanling National Nature Reserve Management Center of Zhejiang, Wenzhou, Zhejiang, China; ^3^ State Key Lab Subtrop Silviculture, Zhejiang A&F University, Hangzhou, Zhejiang, China

**Keywords:** *Euchresta japonica*, UPLC-ESI-MS/MS, comparative metabolomics, KEGG analysis, health-promoting, pharmacological ingredients, differential metabolites

## Abstract

*Euchresta japonica*, a medicinal plant in Chinese herbal medicine, lacks comprehensive metabolite data to explain its health benefits despite its long-standing use. Here, widely targeted metabolome at six different tissues of *E. japonica* was investigated, identifying 2,140 metabolites, including flavonoids, phenolic acids, amino acids, lipids, and alkaloids. Among them, 305 were annotated as key active ingredients, and 364 were active pharmaceutical ingredients for nine human disease-resistance, with 206 co-annotated. Metabolic profiles varied significantly across tissues, with medicinally active metabolites highly concentrated in lateral roots and inflorescences, indicating great medical potential. Notably, the lateral root, rather than the main root, was the primary source of root-derived bioactive metabolites. Additionally, KEGG analysis demonstrated that secondary metabolic pathways, especially “isoflavonoid biosynthesis” and “flavonoid biosynthesis” pathways, played important roles. Overall, lateral roots and inflorescences exhibit the strongest potential for disease treatment, particularly for chronic and multifactorial diseases. This study significantly advances our understanding of *E. japonica*’s chemical composition and underscores its potential as a valuable resource for novel therapeutic applications, providing a strong foundation for further investigation into its pharmacological properties and drug development prospects.

## 1 Introduction

Metabolites, the intermediate and final products of biological processes, are particularly abundant in plants. It is estimated that there are approximately 200,000 distinct metabolites within the plant kingdom, though the majority remain unidentified (Razzaq et al., 2019). These metabolites play crucial roles not only in plant development, growth, and adaptation to biotic and abiotic stresses, but also serve as vital sources of nutrients and medicines for humans. Medicinal plants, which have preventive and therapeutic effects on diseases, are a prime example (Xiao et al., 2022). China boasts a rich diversity of medicinal plants, with over 10,000 species constituting approximately 87% of the total resources of Chinese medicinal materials. The 2020 edition of the Chinese Pharmacopoeia (ChP) has cataloged 499 categories of plant-derived Chinese medicinal materials. The pharmacological efficacy of these medicinal plants is primarily attributed to the biosynthesis of secondary metabolites, which exhibit a wide range of structures and activities (Li et al., 2023).

Plant metabolite profiles can vary greatly depending on species, habitat, growth stage, plant tissues, and other factors. Comprehensive analysis of these metabolites can help assess the quality of medicinal plants and identify health-promoting compounds, which is vital for clinical treatments and drug development. However, identifying plant metabolites is challenging due to the lack of methods capable of profiling the vast array of metabolites simultaneously. Recent rapid advancements in metabolomics technologies have enabled the qualitative and quantitative analysis of small molecular metabolites across different samples, revealing sample-specific differences. Widely targeted metabolomics has emerged as a prevalent method for metabolite analysis in numerous plant species, including medicinal plants such as black sesame (Wang et al., 2018), loquat (Zou et al., 2020), *Trichosanthis radix* (Kang et al., 2020), and citrus herbs (Cao et al., 2023).

The *Euchresta* J. Bonn, a small genus in the Fabaceae family, comprises four species worldwide: *E. japonica* Hook. F. ex Regel, *E. formosana* (Hayata) Ohwi, *E. horsfieldii* (Lesch.) Benn., and *E. tubulosa* Dunn (D). All these species are medicinal plants primarily found in eastern and southeast Asia (Li et al., 2014). The chemical constituents extracted from *Euchresta* species encompass a diverse array of compounds, including flavonoids, alkaloids, and steroids (Li et al., 2014, Li H. et al., 2019; Li W. H. et al., 2019). Modern pharmacological research has demonstrated that *Euchresta* species exhibit a spectrum of biological activities, such as anti-tumor, anti-HIV, anti-platelet aggregation, central nervous system inhibition, blood lipid regulation, and antibacterial properties (Lo et al., 2003; Toda and Shirataki, 2006; Hsu et al., 2007; Kim et al., 2011).

*E. japonica* is an evergreen shrub that grows in shady and humid areas of evergreen broad-leaved forests throughout southern China, Korea, and southwestern Japan. It is also known as Shandougen or Hudoulian in China, and has a rich history of use in traditional Chinese medicine for its pharmacological properties. For example, within the Wu-ling Mountains, the Tujia minority has traditionally employed *E. japonica* to alleviate sore throats and abdominal pain. In Japan, the roots of this species have been utilized for their anti-inflammatory, antiarrhythmic, anticancer, and antiulcer effects (Mizuno et al., 1989). However, due to its inherently low reproductive capacity and slow growth rate, compounded by ongoing habitat destruction and excessive human harvesting, *E. japonica* has been classified as an endangered species (Choi et al., 2013). It is now listed as a second-class protected species in China (2021) and is designated as vulnerable (VU) on the IUCN Red List.

Despite its significant medicinal potential, *E. japonica*’s rarity and endangered status have resulted in limited research, with most studies focusing primarily on its population distribution and genetic conservation (Choi et al., 2013). However, investigations into its metabolite constituents—critical for understanding its pharmacological properties—remain notably scarce. Li et al. (2014) cataloged the metabolites identified in *Euchresta* plants, reporting a total of 86 flavonoids, 14 alkaloids, 4 steroids, 9 other metabolites, and 40 volatile components. Of these, 38 flavonoids (Mizuno et al., 1988a, 1988b, 1992; Shirataki et al., 1980), 11 alkaloids (Ohmiya et al., 1978, 1980), and 1 steroid (Shirataki et al., 1980) were detected in *E. japonica*. Nevertheless, the identified metabolites represent only a small fraction of the thousands potentially present, and the quantities of each remain unknown. Furthermore, the specific metabolites responsible for *E. japonica*’s pharmacological properties have yet to be determined. It is therefore imperative to undertake large-scale metabolite identification in *E. japonica* and to discover the specific metabolites that contribute to its reputed health benefits. Moreover, while the root is the primary part exploited by humans for medicinal purposes, other tissues—such as leaves, stems, inflorescences, and fruits—have scant documentation regarding their use in disease treatment, and their metabolic composition and content remain poorly understood.

In this study, a widely targeted metabolomics approach based on ultra-performance liquid chromatography coupled to triple quadrupole mass spectrometry (UPLC-QQQ-MS) was used to systematically identify and quantify metabolites across various tissues of *E. japonica*. Additionally, we pinpointed the key ingredients within these tissues that possess health-promoting properties. More importantly, metabolite content comparison, hierarchical clustering, and metabolic pathway enrichment analyses were also performed to identify the differential functional components that may be associated with the bioactivity and health benefits specific to distinct tissues of *E. japonica*. This study significantly enhances our knowledge of the chemical components, metabolomic profiles, and health-promoting function of the entire *E. japonica* plant. Furthermore, it lays a robust theoretical foundation that can inform the future development and application of this medicinal species.

## 2 Materials and methods

### 2.1 Plant materials

The *E. japonica* plants used in this study were cultivated in a greenhouse for two years and then transplanted to the wild for one year. During the fruiting period, twelve healthy, mature plants of similar age and growth conditions were selected from Wuyanling Nature Reserve, Zhejiang Province (27°41′37.54″N, 119°40′48.52″E), China. Fresh samples of main roots (MR), lateral roots (LR), stems (S), leaves (L) and black fruits (Fr) were collected from these plants. Inflorescences (Inf) were collected from three-year-old cultivated plants at full flowering stage. All samples were immediately frozen in liquid nitrogen and stored at –80°C for RNA and metabolic extraction. Experiments were conducted with four biological replicates.

### 2.2 Sample preparation and extraction

Metabolite extraction, detection, and quantitative analysis for the 24 samples were carried out using a widely targeted metabolomics approach by Metware Biotechnology Co., Ltd. (Wuhan, China). The samples were freeze-dried with a lyophilizer (Scientz-100F) and ground into fine powder with a grinder (MM400, Restc) at 30 Hz for 1.5 min. For each sample, 50 mg of powder was weighted and dissolved in 1.2 mL of pre-cooled (–20°C) 70% methanolic aqueous internal standard extract. The solution was vortexed every 30 min for 30 sec, repeated six times. After centrifugation at 12,000 rpm for 3 min, the extracts were filtrated through a 0.22 μm pore size microporous membrane and stored in injection vials for UPLC-MS/MS analysis.

### 2.3 Widely targeted metabolomic analysis

#### 2.3.1 UPLC conditions and ESI-Q TRAP-MS/MS

Then, an UPLC-ESI-MS/MS system (UPLC, ExionLC™ AD, https://sciex.com.cn/) and Tandem mass spectrometry system (https://sciex.com.cn/) were used to analyze the sample extracts. Each parameter choice was carefully considered based on established scientific principles and previous research in the field (Li et al., 2022; Cao et al., 2023). The UPLC analytical conditions were set as follows: (1) Column: Agilent SB-C18 (1.8 μm, 2.1 mm * 100 mm); (2) Mobile phase: Solvent A, pure water with 0.1% formic acid; Solvent B, acetonitrile with 0.1% formic acid; (3) Gradient elution: The analysis began with 95% A and 5% B. Over 9 min, a linear gradient was applied to reach 5% A and 95% B, which was maintained for 1 min. Then, the composition was adjusted to 95% A and 5.0% B over 1.1 min and held for 2.9 min; (4) Flow velocity, 0.35 mL/min; column oven, 40°C; injection volume, 2 μL. The effluent was alternately connected to an ESI-triple quadrupole-linear ion trap (Q-TRAP)-MS.

The ESI source operation parameters were set as follows: Source temperature, 500°C; Ion spray voltage (IS), 5500 V (positive ion mode)/-4500 V (negative ion mode); Ion source gas I (GSI), II (GSII), and curtain gas (CUR) were set at 50, 60, and 25 psi, respectively; Collision-activated dissociation (CAD) was set to high. QQQ scans were acquired as multiple reaction monitoring (MRM) experiments with collision gas (nitrogen) set to medium. Declustering potential (DP) and collision energy (CE) for individual MRM transitions were optimized as needed. Specific MRM transitions were monitored based on the metabolites eluted during each period.

#### 2.3.2 Qualitative and quantitative determination of metabolites

For qualitative analysis, the secondary mass spectrum data from UPLC-MS/MS were acquired and pre-processed using Analyst software (Version 1.6.3). To ensure the accurate metabolite annotation, isotopic signals, duplicate signals containing K^+^, Na^+^, and NH_4_^+^ ions, and repeated fragment ions from other larger molecular weight substances were excluded from the analysis. The data were then annotated using the self-built Metware database (MWDB, Metware Biotechnology Co., Ltd., Wuhan, China).

For quantitative analysis, the MRM mode of triple-quadrupole mass spectrometry was employed. Peak detection, integration and correction were performed using MultiaQuant™ software. The corrected peak area for each chromatographic peak was used as an indicator of the relative concentration of the metabolites.

### 2.4 Network pharmacology

#### 2.4.1 Identification of the key active ingredients in traditional Chinese medicines in *Euchresta japonica*

All metabolites of *E. japonica* identified through widely targeted metabolomics were queried in the Traditional Chinese Medicine Systems Pharmacology Database and Analysis Platform (TCMSP, https://old.tcmsp-e.com/tcmsp.php). Metabolites meeting the criteria of oral bioavailability (OB) ≥ 5% and drug-likeness (DL) ≥ 0.14 (Li et al., 2022) were considered key bioactive ingredients in Traditional Chinese Medicines derived from *E. japonica*. The related-targets and related-diseases information of these identified metabolites were listed as the TCMSP database annotation.

#### 2.4.2 Identification of the active pharmaceutical ingredients for human diseases-resistance

Based on the disease-related annotation in the TCMSP database, the active pharmaceutical ingredients in *E. japonica* with function of anti-cancer/tumor, anti-Alzheimer’s disease, analgesics, anti-myocardial infarction, anti-inflammatory, anti-arthritis, anti-gestational hypertension, anti-stroke, and anti-cardiovascular disease were summarized. The identification process of these active pharmaceutical ingredients followed previous methodologies with some modifications (Li et al., 2022; Xia et al., 2023).

### 2.5 Differential metabolites analysis

Multivariate statistical analysis methods were employed to analyze the metabolic data of *E. japonica*. Unsupervised principal component analysis (PCA) was performed using prcomp function in R (www.r-project.org), with data scaled to unit variance before analysis. Hierarchical Cluster Analysis (HCA) and Pearson Correlation Coefficients (PCC) were conducted using the R package Complex-Heatmap. HCA results for samples and metabolites were displayed as heatmaps with dendrograms, while PCC between samples were presented heatmaps only.

To identify the differential metabolites, Orthogonal Partial Least Squares-Discriminant Analysis (OPLS-DA) was used to maximize the differences between metabolite profiles of two samples (Thévenot et al., 2015). The Variable Importance in Projection (VIP) scores from the OPLS-DA model were used for preliminary screening of differential metabolites. Significantly differentially expressed metabolites (DEMs) were determined by VIP (VIP ≥ 1) and absolute Log2 fold change (|Log2FC| ≥ 1.0, *p* ≤ 0.05). To prevent overfitting, a permutation test with 200 permutations was conducted. Identified metabolites were annotated using KEGG Compound database (http://www.kegg.jp/kegg/compound/) and MetMap databases, and annotated metabolites were mapped to KEGG Pathway database (http://www.kegg.jp/kegg/pathway.html) and MetMap databases. Pathways with significantly regulated metabolites were subjected to metabolite sets enrichment analysis (MSEA), and significance was determined by *p*-values from the hypergeometric test.

## 3 Results and discussion

### 3.1 Identification and overview of metabolites in six tissues of *Euchresta japonica*

**Figure 1:**
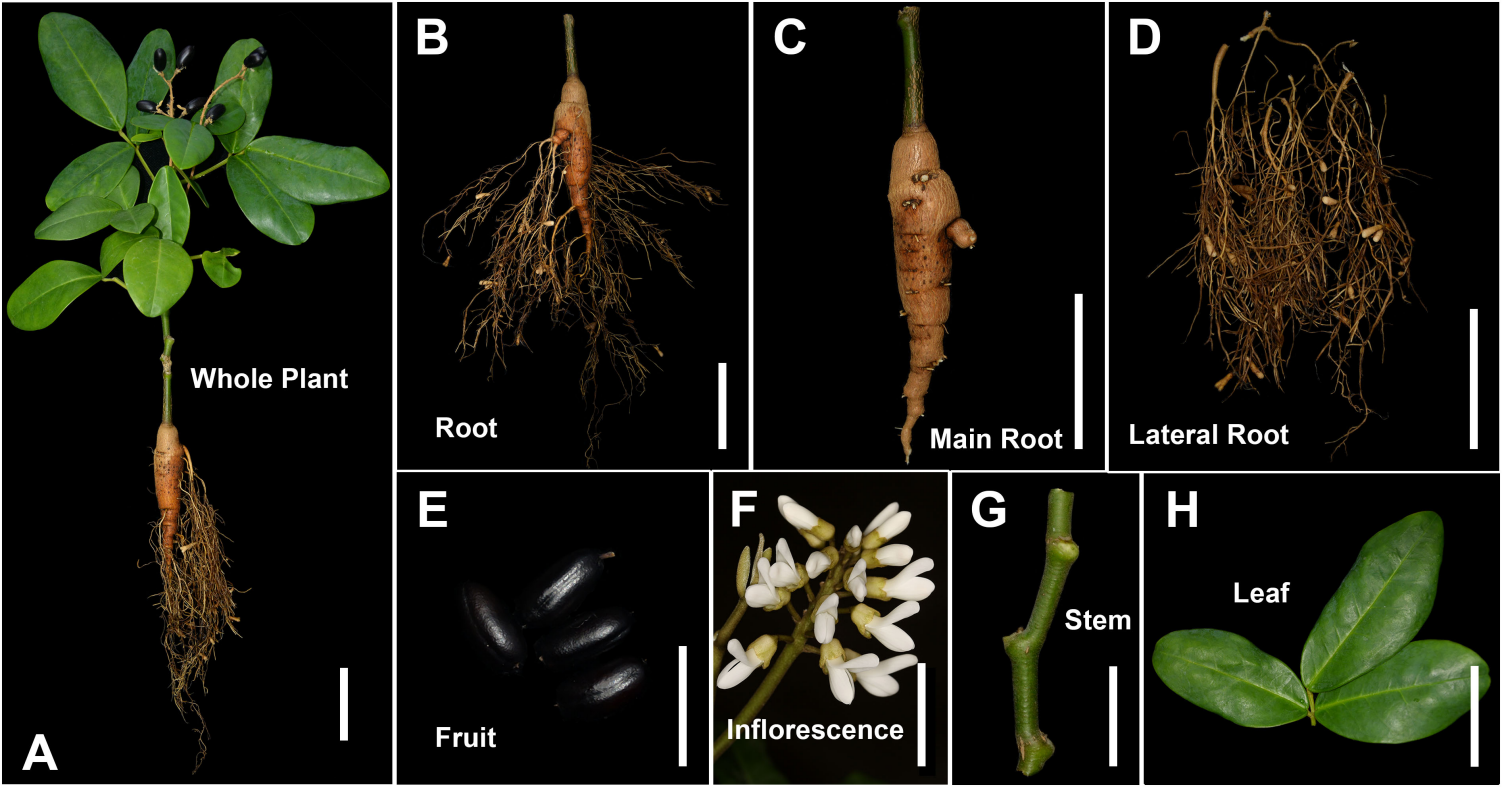
Different tissues of *Euchresta japonica* plant. **(A-H)** whole plant, root, main-root (MR), lateral-root (LR), fruit (Fr), inflorescence (Inf), stem (S), leaf (L). Scale bars, 5 cm in **(A-D)**, **(F, H)**, 2 cm in **(E, G)**.

To investigate metabolites in *E. japonica*, we used a widely targeted metabolite method based on UPLC-ESI-MS/MS on samples from different tissues, including MR, LR, Fr, Inf, S, and L (**Figure 1**). The good reproducibility and reliability of UPLC-ESI-MS/MS analysis were demonstrated by the highly overlapping total ion current (TIC) chromatograms of the Quality Control (QC) samples (**Supplementary Figure S1**).

**Figure 2:**
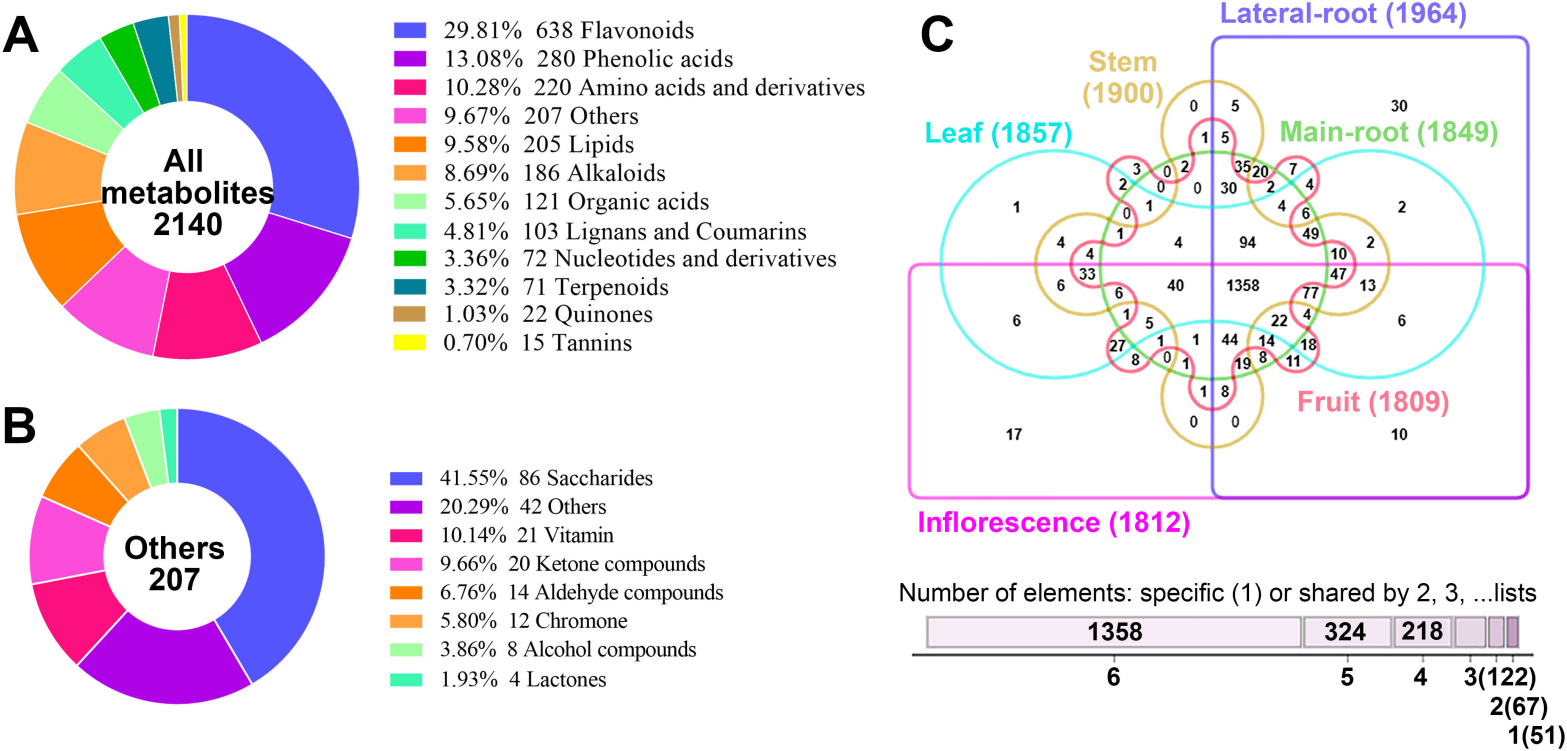
Analysis of the 2140 metabolites identified from the six tissues of *Euchresta japonica*. **(A)** Classification of the 2140 metabolites. **(B)** Classification of the 207 metabolites belonging to the “others” category. **(C)** Venn diagram showed the distribution of 2140 metabolites in the six tissues of *Euchresta japonica*.

A total of 2,140 metabolites were identified from six tissues of *E. japonica*, which can be classified into twelve distinct categories: flavonoids (638, 29.81%), phenolic acids (280, 13.08%), amino acids and derivatives (220, 10.28%), lipids (205, 9.58%), alkaloids (186, 8.69%), organic acids (121, 5.65%), lignans and coumarins (103, 4.81%), nucleotides and derivatives (72, 3.36%), terpenoids (71, 3.32%), quinones (22, 1.03%), tannins (15, 0.7%), and others (207, 9.67%) (**Figure 2A**; **Supplementary Table S1**). Furthermore, several categories were further subdivided into subclasses (**Supplementary Figure S2**). Specifically, the 207 other compounds comprised eight subclasses: 86 saccharides, 21 vitamins, 20 ketone compounds, 14 aldehyde compounds, 12 chromones, 8 alcohol compounds, 4 lactones, and 42 others (**Figure 2B**). To our best knowledge, this is the first comprehensive identification of metabolites across different tissues of *E. japonica*. Overall, the number of metabolites was far greater than that detected in most other plants (Li et al., 2022; Cao et al., 2023; Xia et al., 2023). This finding underscores the efficacy of the UPLC-ESI-MS/MS-based widely targeted metabolomics approach for the comprehensive identification of metabolites in plants.

The metabolites in the six tissues were also categorized, revealing that the proportions of metabolites across twelve categories were broadly similar among different tissues, with the top five being flavonoids, phenolic acids, amino acids and derivatives, lipids, and alkaloids (**Supplementary Figure S3**). Although all twelve categories were detected in all six tissues, their relative levels varied significantly. Correlation analysis showed similar profiles between MR and LR (0.42≤|r|≤0.49), MR and S (0.39≤|r|≤0.47), L and Inf (0.36≤|r|≤0.51) (**Supplementary Figure S4**). Each tissue contained over 1800 metabolites, with LR having the most (1964) and Fr the least (1801) (**Figure 2C**). Any two tissues shared over 1600 metabolites, with the highest overlap (over 1750) between any pair of MR, LR, and S. A total of 1358 metabolites were identified across all six tissues, while 51 were tissue-specific. Notably, 30 of these were in LR and 17 in Inf. Furthermore, 18 of the 51 unique metabolites were alkaloids (35.29%), much higher than the proportion in total metabolites (186/2140, 8.69%). These findings indicate both conservation and diversity of metabolites across different tissues of *E. japonica*.

Additionally, we assessed which tissue had the highest accumulation of each metabolite. Results showed that LR and Inf contained the highest number of richest metabolites, 583 (27.24%) and 552 (25.79%), respectively, followed by L (416, 19.44%), Fr (270, 12.62%), MR (166, 7.76%) and S (153, 7.15%) (**Supplementary Figure S5A**, **Supplementary Table S1**). Statistical analysis of the distribution of richest metabolites across the six tissues also revealed notable differences in certain metabolites’ accumulation patterns (**Supplementary Figures S5B–M**).

### 3.2 Screening the key active ingredients related to human health

For a long time, *E. japonica*, as a rare traditional Asian herbal medicine, has been used in folk remedies to treat conditions such as enteritis, diarrhea, pain (abdominal, stomach, toothache), and even cancers like throat and esophageal cancer (Mizuno et al., 1989). However, its potential health-promoting active components remain largely unclarified. To address this, we used the TCMSP database to screen the identified metabolites for key function-active components in *E. japonica*.

Among the 2,140 metabolites examined, 596 were identified as chemical constituents of traditional Chinese medicines. These included 219 flavonoids, 82 phenolic acids, 41 organic acids, 40 alkaloids, 33 lipids, 31 lignans and coumarins, 28 terpenoids, 25 amino acids and derivatives, 19 nucleotides and derivatives, 7 quinones, 7 tannins, and 64 other compounds (**Supplementary Table S2**). Among them, 402 were associated with at least one target protein and disease, 7 only with target proteins, and the remaining 187 had no corresponding target proteins or diseases.

**Figure 3:**
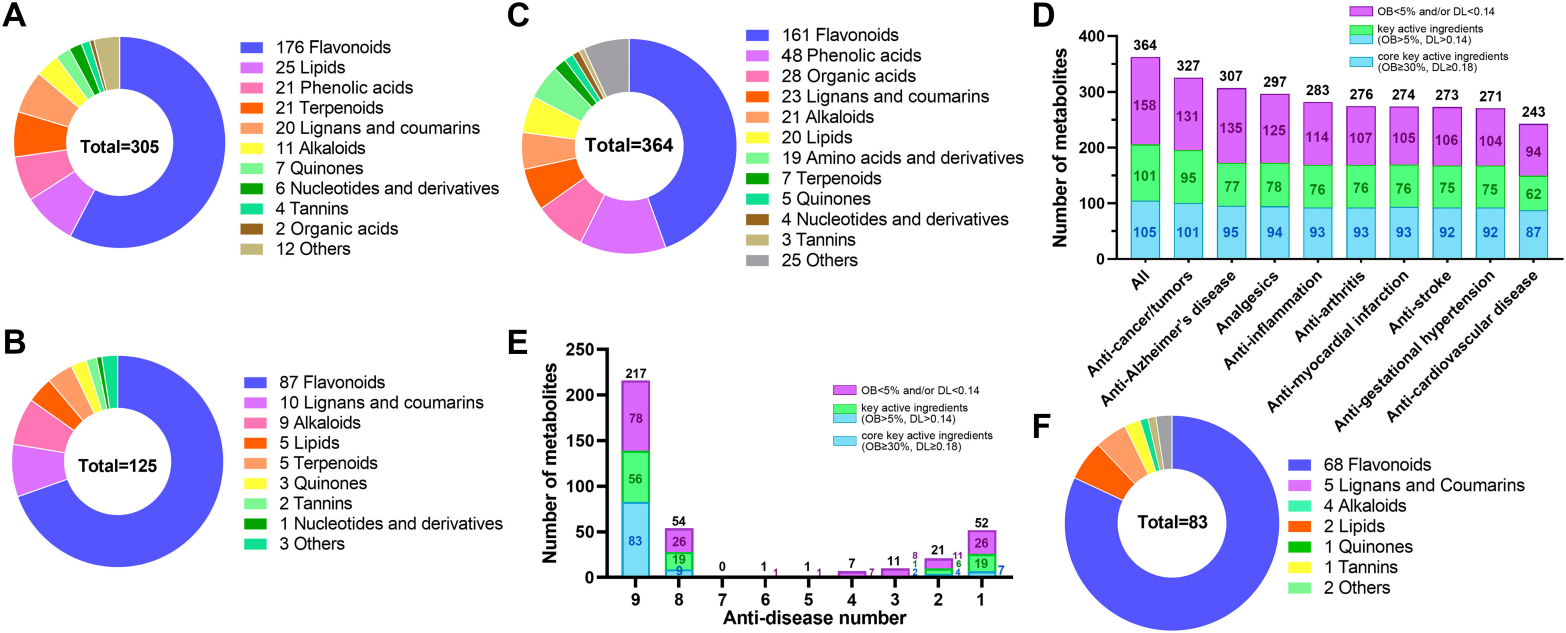
Identification of 305 key active ingredients, 125 core key active ingredients and 83 active pharmaceutical ingredients in *Euchresta japonica*. The ring chart showed the classification of the 305 key active ingredients **(A)** and 125 core key active ingredients **(B)**. **(C)** The ring chart showed the classification of the 364 metabolites that correspond to at least one of the nine diseases. **(D)** The number of metabolites corresponding to the nine diseases. **(E)** The bar chart showed the anti-disease number of the 364 metabolites. **(F)** The ring chart showed the classification of the 83 core key active pharmaceutical ingredients for nine major diseases resistance. OB, oral bioavailability; DL, drug-likeness. Key active ingredient, meet the criteria (OB ≥ 5% and DL ≥ 0.14); Core key active ingredient, meet the criteria (OB ≥ 30% and DL ≥ 0.18); Core key active pharmaceutical ingredient: core key active ingredient associated with at least one disease.

To further characterize key active ingredients, we used OB ≥ 5% and DL ≥ 0.14 as screening criteria (Li et al., 2022). Consequently, 305 of 596 metabolites met these criteria (**Figure 3A**; **Supplementary Table S2**). Over half (176, 57.5%) were flavonoids. The rest included 25 lipids, 21 phenolic acids, 21 terpenoids, 20 lignans and coumarins, 11 alkaloids, 7 quinones, 6 nucleotides and derivatives, 4 tannins, 2 organic acids, and 12 others. Moreover, 125 of the 305 key active ingredients met a stricter criterion (OB ≥ 30% and DL ≥ 0.18) for screening potential drug candidates (Jiang et al., 2021; Qu et al., 2021). These 125 metabolites were identified as core key active ingredients and composed of 87 flavonoids, 10 lignans and coumarins, 9 alkaloids, 5 lipids, 5 terpenoids, 3 quinones, 2 tannins, 1 nucleotides and derivatives, and 3 other compounds (**Figure 3B**). These results indicate that flavonoids are the primary chemical metabolites contributing to *E. japonica*’s health-promoting functions. However, other metabolites such as lipids, phenolic acids, terpenoids, lignans and coumarins, alkaloids, quinones may also make important roles.

Among the 305 key active ingredients, 212 were associated with at least one disease and/or target protein, while the remaining 93 were not. These 212 were found to play roles in interacting with 421 target proteins (**Supplementary Table S3**) and corresponded to 370 diseases (**Supplementary Table S4**). Additionally, 110 of the 125 core key active ingredients meeting potential drug-selection criteria were linked to 227 target proteins and corresponded to 313 diseases (**Supplementary Tables S3**, **S4**). The diseases primarily involved various cancers, e.g. breast, colorectal, lung, bladder, prostate, and ovarian cancers. Other diseases encompassed Alzheimer’s disease, analgesics, tumors, inflammation, arthritis, gestational hypertension, myocardial infarction, stroke, cardiovascular disease, and so on (**Supplementary Tables S3**–**S5**). Together, these findings highlight that the identified metabolites are crucial or core key active ingredients for human health in *E. japonica*.

Furthermore, among the 291 non-key active ingredients not meeting the OB ≥ 5% and DL ≥ 0.14 criteria, 196 were associated with targets and/or diseases, corresponding to 126 different target proteins and 359 different target diseases. Many were related to the aforementioned diseases and additional diseases, e.g., depression, Parkinson’s disease, Schizophrenia, alcoholism, anxiety disorders, and insomnia. (**Supplementary Table S6**). Notably, of the 187 metabolites with no identified corresponding target proteins or diseases, 93 met the OB ≥ 5% and DL ≥ 0.14 criteria. Among them, 15 (8 flavonoids, 2 alkaloids, 2 terpenoids, 2 lignans, and 1 lipid) satisfied the potential drug-candidate thresholds (**Supplementary Table S2**). These results suggest that some metabolites lacking identified target proteins and diseases in the TCMSP database may still play significant health-promoting roles and could be valuable for new drug development.

### 3.3 Identification of the active pharmaceutical ingredients for nine major diseases-resistance in *Euchresta japonica*

The nine human diseases—cancers/tumors, Alzheimer’s disease, analgesics, inflammation, arthritis, myocardial infarction, stroke, gestational hypertension, and cardiovascular diseases—pose global health threats. Based on previous metabolite annotation, these diseases are key targets of *E. japonica* metabolites. To identify the active pharmaceutical ingredients against these nine major diseases in *E. japonica*, we queried 596 metabolites confirmed as traditional Chinese medicine chemical components in TCMSP. Among them, 364 were associated with at least one of the nine diseases (**Supplementary Table S2**), including 161 flavonoids, 48 phenolic acids, 28 organic acids, 23 lignans and coumarins, 21 alkaloids, 20 lipids, 19 amino acids and derivatives, etc. (**Figure 3C**; **Supplementary Table S2**). Specifically, 327 were related to anti-cancer/tumors, 307 to anti-Alzheimer’s disease, 297 to analgesics, 283 to anti-inflammation, 276 to anti-arthritis, 274 to anti-myocardial infarction, 273 to anti-stroke, 271 to anti-gestational hypertension, and 243 to anti-cardiovascular diseases (**Figure 3D**; **Supplementary Table S2**). Moreover,206 (56.59%) of the 364 were annotated as key active ingredients, including 105 (28.85%) core key active ingredients (**Figure 3D**; **Supplementary Table S2**). Notably, many metabolites were associated with multiple diseases. About 60% (217/364) were effective against all nine diseases, with 83 being core key active ingredients, 68 of which were flavonoids (**Figures 3E, F**; **Supplementary Table S2**). These findings partially explain why *E. japonica* is widely used in folk medicine for treating various diseases, from small ailments like stomach pain to intractable diseases such as various cancers. The extensive identification of metabolites with health-promoting potential highlight *E. japonica*’s promise for new herbal medicine development.

### 3.4 Identification of differential metabolites

#### 3.4.1 Data quality assessment

**Figure 4:**
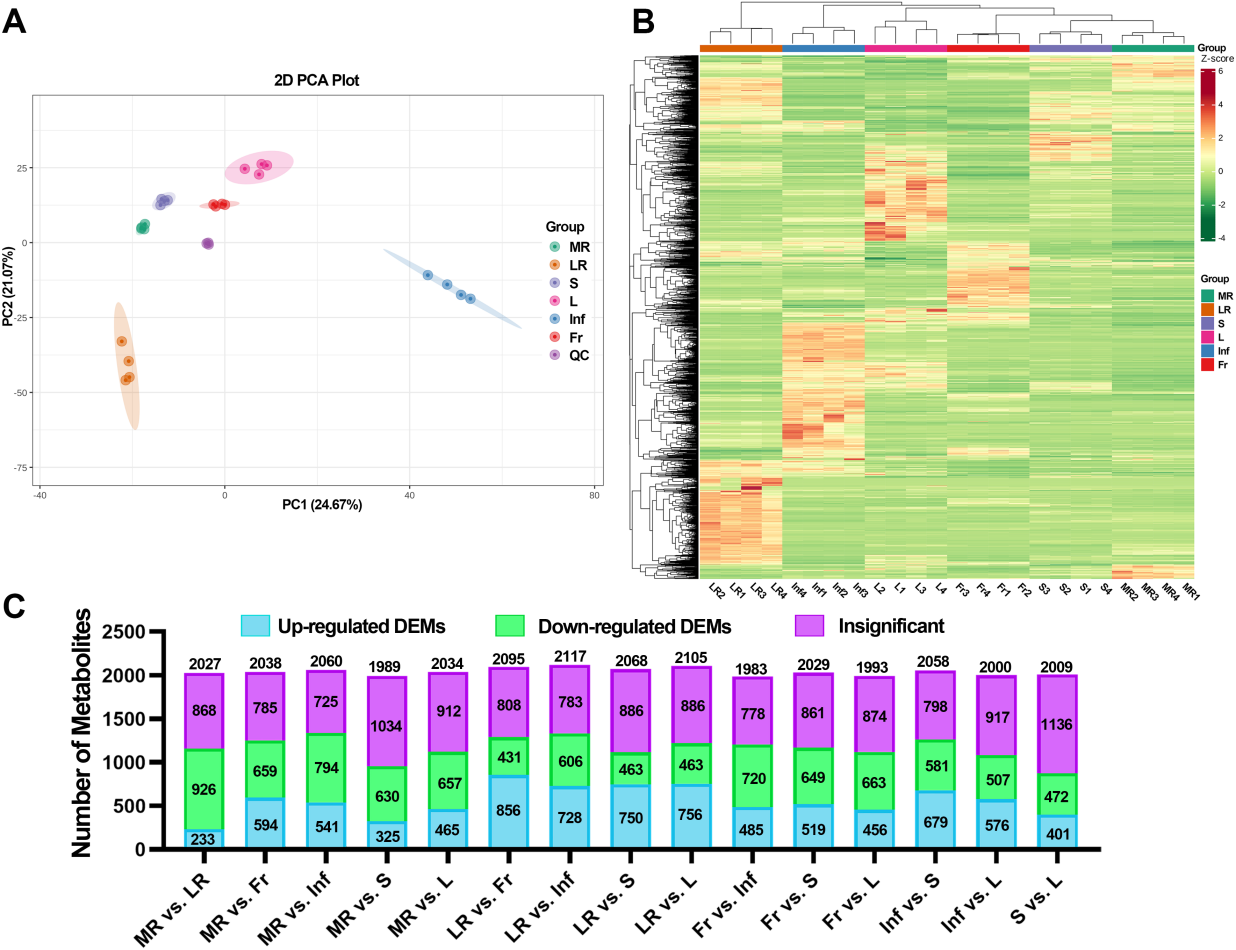
Different metabolome profiling of six tissues of *Euchresta japonica*. **(A)** The result of principal component analysis (PCA). **(B)** The hierarchical cluster analysis (HCA) of metabolites. **(C)** Statistical analysis of the numbers of up/down-regulated metabolites in 15 comparison groups. MR, LR, Fr, Inf, S, and L represent main-roots, lateral-roots, fruits, inflorescence, stems, and leaves of *E. japonica*, respectively.

To identify differential metabolites among different *E. japonica* tissues, the PCA and HCA were performed. The PCA results indicated clear separation among tissue groups. High aggregation of QC samples confirming the stability and reliability of the chromatography and quality detection system. As shown in **Figure 4A**, the two principal components (PC1 and PC2) explained 45.74% of the cumulative variance (PC1: 24.67%, PC2: 21.07%). Notably, PC1 and PC2 primarily differentiated the Inf and LR from the other four tissues. The remaining tissues, MR, S, L, and Fr were relatively clustered but still distinguishable along PC1 and PC2. These results suggest that the metabolites in *E. japonica* tissues differ significantly, with Inf and LR showing the most pronounced differences.

In line with the PCA results, HCA also demonstrated significant tissue-specific metabolite differences (**Figure 4B**). In PCA, the four biological replicates of each tissue clustered tightly, and the correlation analysis showed that the Pearson correlation coefficient |r| was close to 1 for all inter-group comparisons (**Supplementary Figure S4**), confirming high data reproducibility.

#### 3.4.2 OPLS-DA analysis

OPLS-DA was used to screen differential metabolites among tissues. The OPLS-DA models exhibited high predictability (Q^2^ ≥ 0.991) and strong goodness of fit (R^2^X ≥ 0.722, R^2^Y = 1) across all fifteen pairwise comparisons among the six tissues, indicating model stability and reliability (**Supplementary Figure S6**).

The OPLS-DA model score plots (**Supplementary Figure S7**) revealed significant separations among tissue groups, suggesting distinct metabolite profiles. The OPLS-DA S-plots, which graphically project specific metabolites, showed that red and green dots represent metabolites with VIP values ≥ 1 and < 1, respectively (**Supplementary Figure S8**). The top 20 metabolites with higher VIP values for each comparison were shown in **Supplementary Figure S9**. Notably, in LR comparisons (except LR vs. Inf), at least 15 of the top 20 metabolites were upregulated in LR compared to other tissues.

Fold change (FC) values were calculated to provide a clearer and more intuitive representation of overall metabolic differences. A dynamic distribution map of metabolite content differences was created, highlighting the top 10 up- and down-regulated metabolites (**Supplementary Figure S10**). Each pairwise comparison identified more than 1,100 metabolites meeting the criteria of |Log2FC| ≥ 1.0, with the most (1,543) in LR vs. L and the fewest (1,127) in MR vs. S (**Supplementary Table S7**). Moreover, among the 15 comparisons, LR vs. MR/Fr/Inf/S/L exhibited a higher number of up-regulated metabolites, with 1,056, 974, 825, 843, and 885 metabolites, respectively (**Supplementary Table S7**).

To further explore tissue differences, DEMs were screened from the 2140 identified metabolites based on FC and VIP scores. Using the criteria (VIP ≥ 1; |Log2FC| ≥ 1.0; *p* ≤ 0.05), a total of 2045 DEMs were identified in at least one pairwise comparison, accounting for 97.2% of all detected metabolites. As shown in **Figures 4C**, **Supplementary Figure S11** and **Supplementary Tables S7**, **S8**, over 40% of detected metabolites in all pairwise comparisons were DEMs, with the most (1,335) in MR vs. Inf and the fewest (873) in S vs. L. These results strongly suggest significant variations in metabolite composition and regulation among tissues.

#### 3.4.3 K-means clustering algorithm analysis

**Figure 5:**
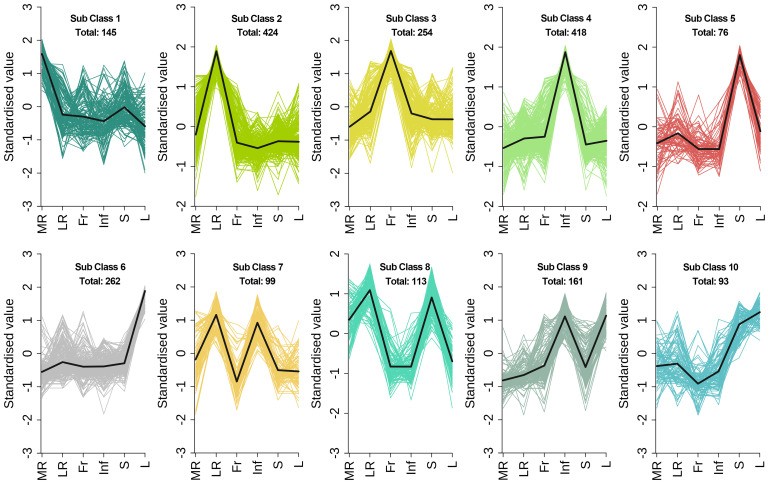
K-means clustering algorithm analysis of differential metabolites in different tissues of *Euchresta japonica*. The x axis represented the different tissues of *E. japonica*, and the y axis represented the standardized content of per metabolite. MR, LR, Fr, Inf, S, and L represent main-roots, lateral-roots, fruits, inflorescence, stems, and leaves, respectively. The total number of differential metabolites per subclass was shown.

To evaluate metabolite variations across tissues of *E. japonica*, we performed unit variance scaling on 2045 DEMs and clustered them into 10 subclasses using K-means analysis (**Figure 5**; **Supplementary Table S7**). This classification revealed tissue-specific metabolite accumulation patterns. For instance, subclass 1 to 6 exhibited high metabolite accumulation in MR, LR, Fr, Inf, S and L, respectively, with 145, 424, 254, 418,76 and 262 DEMs each (**Figure 5**; **Table 1**). Saccharides, though a minor fraction of all DEMs, were prominent in MR-specific subclass 1(**Figure 5A**). Flavonoids dominated subclasses 2 to 6, indicating their prevalence in LR, Fr, Inf, S, and L (**Figures 5B–F**; **Table 1**). Subclass 2 was distinguished by elevated alkaloids (35) and terpenoids (25), with lipids and phenolic acids ranking second to subclass 4. This indicates that flavonoids, phenolic acids, lipids, alkaloids, and terpenoids are the primary metabolites with high accumulation in the lateral roots of *E. japonica*. Subclass 3 was rich in flavonoids, amino acids and derivatives, and organic acids. Subclass 4, with 418 Inf-concentrated metabolites, included 104 flavonoids, 83 lipids, 65 phenolic acids, 45 amino acids and derivatives, and 32 organic acids, totally accounting for 78.7%. Subclass 5, with 76 stem-enriched metabolites, highlighted flavonoids, phenolic acids, and alkaloids. Subclass 6, boasting 262 leaf-enriched metabolites, featured flavonoids, phenolic acids, and saccharides. Subclasses 7 to 10 had elevated metabolite levels in tissue pairs: LR & Inf (99), LR & S (113), Inf & L (161), and S & L (93), with flavonoids being predominant. Overall, inflorescences and lateral roots of *E. japonica* had the highest number of metabolites at elevated levels, followed by leaves, stems, fruits, and main roots.

**Table 1:** The number of different types of DEMs in the 10 subclasses of K-means clustering analysis.

Sub Class Types	Sub Class 1	Sub Class 2	Sub Class 3	Sub Class 4	Sub Class 5	Sub Class 6	Sub Class 7	Sub Class 8	Sub Class 9	Sub Class 10	Total
Alkaloids	17	35	23	21	10	16	14	6	13	9	164
Amino acids and derivatives	31	18	42	45	7	18	23	7	10	6	207
Flavonoids	8	160	59	104	24	121	8	30	78	37	629
Lignans and Coumarins	3	19	12	9	5	13	4	24	4	6	99
Lipids	11	47	13	83	0	6	20	2	7	1	190
Nucleotides and derivatives	8	9	15	16	2	3	12	5	1	0	71
Organic acids	5	12	30	32	4	12	2	2	12	5	116
Phenolic acids	16	61	21	65	18	27	9	27	7	20	271
Quinones	0	9	8	0	0	2	0	1	1	1	22
Tannins	0	6	0	0	2	1	0	1	1	1	12
Terpenoids	6	25	7	12	3	5	1	2	6	3	70
Saccharides	32	1	9	10	0	21	1	2	9	0	85
Others	8	22	15	21	1	17	5	4	12	4	109
Total	145	424	254	418	76	262	99	113	161	93	2045

### 3.5 Differences in the chemical constituents among the different tissues of *Euchresta japonica*

Among the 2045 DEMs, there were 629 flavonoids, 271 phenolic acids, 207 amino acids and derivatives, 190 lipids, 164 alkaloids, 116 organic acids, 99 lignans and coumarins, 85 saccharides, 71 nucleotides and derivatives, 70 terpenoids, 22 quinones, 12 tannins, and 194 others (**Table 1**; **Supplementary Table S7**). Detailed information on each metabolite category in the different comparisons was provided in **Supplementary Table S8**. The tissues with the richest metabolites were listed in **Supplementary Table S1** and illustrated in **Supplementary Figure S5**. The characteristics and differences in the chemical composition were shown in **Supplementary Figures S12–24**.

#### 3.5.1 Flavonoids

Flavonoids are the most abundant metabolites in *E. japonica*, with 638 identified across six tissues. They can be further categorized into nine subclasses: flavones, isoflavones, flavonols, flavanones, chalcones, dihydroisoflavones, flavanols, flavanonols, and others (**Supplementary Figure S2A**). Each tissue of *E. japonica* identified 82.1% to 87.9% of total flavonoids, with the highest proportion found in LR, followed by L, Inf, S, and MR (**Supplementary Figure S12**). Similarly, LR also contained the highest number of richest flavonoids (30.4%, 194), followed by L and Inf (**Supplementary Figure S5B**). Comparative analyses revealed more up-regulated flavonoids in LR, L, and Inf than in other tissues, while MR contained more down-regulated ones (**Supplementary Table S8**). In summary, although the number of detectable flavonoid metabolites varied little among the six tissues, the concentration of flavonoids in LR, L, and Inf was significantly higher than that in Fr, S, and MR, highlighting their potential in *E. japonica*’s medicinal value.

**Figure 6:**
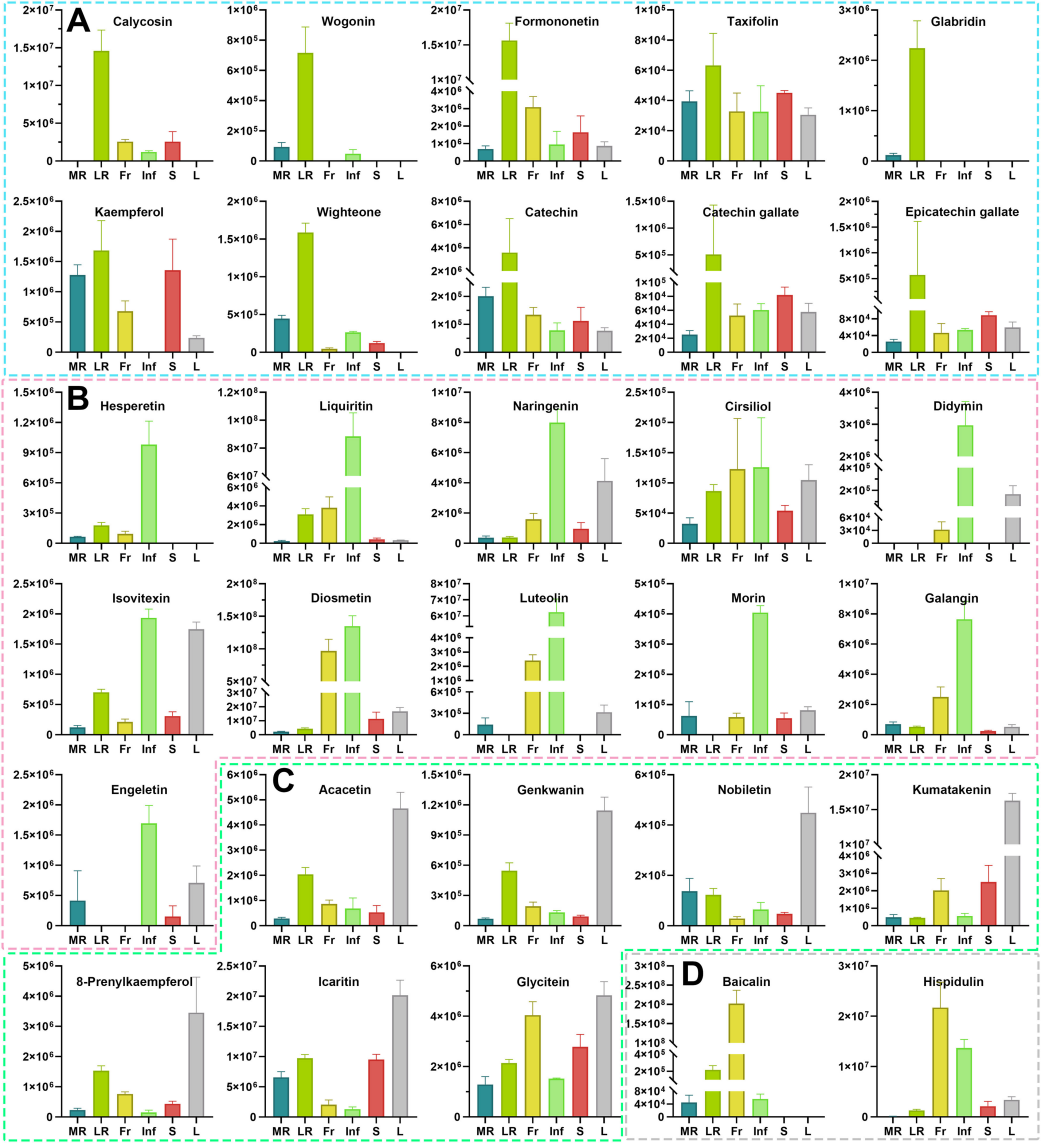
The relative contents of the 30 health-promoting flavonoids metabolites in different tissues of *Euchresta japonica*. More health-promoting flavonoid metabolites accumulate at high levels in the LR, Inf and L. Flavonoids accumulated the highest amount in LR **(A)** Calycosin; Wogonin; Formononetin; Taxifolin; Glabridin; Kaempferol; Wighteone; Catechin; Catechin gallate; Epicatechin gallate), Inf **(B)** Hesperetin; Liquiritin; Naringenin; Cirsiliol; Didymin; Isovitexin; Diosmetin; Luteolin; Morin; Galangin; Engeletin), L **(C)** Acacetin; Genkwanin; Nobiletin; Kumatakenin; 8-Prenylkaempferol; Icaritin; Glycitein), and Fr **(D)** Baicalin; Hispidulin). Each relative content value was mean (± SD) of four biological replicates. MR, LR, Fr, Inf, S, and L represent main-roots, lateral-roots, fruits, inflorescence, stems, and leaves, respectively.

Flavonoids are well-documented for their significant role in managing chronic diseases by inhibiting oxidative damage and persistent inflammation (Ding et al., 2022). Pharmacological data further underscore the importance of flavonoids in disease resistance (**Supplementary Table S9**). Among the 87 core key active flavonoids, 68 were effective against nine major diseases, many highly accumulated in LR, Inf, and L (**Supplementary Tables S2**, **S9**). Many of these flavonoids are known for their antioxidant, anti-inflammatory, anti-arteriosclerotic, anti-tumor, and cancer therapeutic effects. For instance, wogonin, a flavone with notable antitumor activity and a potential chemosensitizer in cancer therapy (Huynh et al., 2017, 2020; Zhang et al., 2022), was highly concentrated in LR but low in MR/Inf and absent in Fr/S/L (**Figure 6A**). Calycosin and formononetin, isoflavones with strong cardiovascular protective effects (Chen et al., 2022; Ma et al., 2022; Qian et al., 2024), were most abundant in LR, followed by Fr or S (**Figure 6A**). Other flavonoids, such as taxifolin (Topal et al., 2016), glabridin (Parlar et al., 2020), kaempferol (Devi et al., 2015), wighteone (Wang et al., 2024), catechin, catechin gallate and epicatechin gallate (Musial et al., 2020), which exhibit excellent antioxidant and/or anti-inflammatory effects, were also highly accumulated in LR (**Figure 6A**). Similarly, several core key active flavonoids with the highest accumulation in Inf have also been reported to have diverse physiological and pharmacological effects. These include four flavanones (hesperetin, liquiritin, naringenin, cirsiliol, and didymin), three flavones (isovitexin, diosmetin, and luteolin), two flavonols (morin and galangin), and one flavanonol (engeletin) (**Figure 6B**). Isovitexin is a potential inhibitor of multiple tumor cells, including colon, breast, ovarian, prostate, esophageal, and pancreatic cancer, demonstrating its capability for promoting apoptosis in tumor cells (Yang et al., 2013; Ganesan and Xu, 2017; Ghanbari-Movahed et al., 2023). Engeletin, with anti-inflammatory, antioxidant, and immunomodulatory properties (Zhong et al., 2023), is a promising drug candidate. Flavonoids with higher relative contents in L include acacetin, genkwanin, nobiletin, kumatakenin, 8-prenylkaempferol, icaritin, glycitein, etc. (**Figure 6C**). Acacetin has demonstrated anti-inflammatory, antiperoxidative, and anticancer activities, and is a promising potential drug for treating osteoarthritis (Kim et al., 2014; Chen et al., 2020). Icaritin possesses a wide range of pharmacological effects, including alleviating sexual dysfunction, osteoporosis, cardiovascular diseases (Reyes-Hernández et al., 2024), and showing efficacy against solid tumor, particularly advanced hepatocellular carcinoma (Luo et al., 2024). Among the 11 core key active flavonoids with the highest accumulation in Fr, baicalin and hispidulin (**Figure 6D**) are noted for their anti-diabetic, anti-inflammatory, antioxidant, and anticancer properties (Wang et al., 2020; Miao et al., 2024). The six core key active flavonoids with the highest accumulation in S include three flavanols (cinchonain Ic, cinchonain Id, and garbanzol), two isoflavones (flemiphilippinin C, and glycyroside), and one other flavonoid (kushenol O) (**Supplementary Table S1**). Taken together, many flavonoids with diverse pharmacological activities like anti-cancer/tumor, anti-inflammatory, etc., are predominantly concentrated in LR, Inf, and L, and those flavonoids can be used as lead compounds for the development of potential therapeutic agents. For example, wogonin with high anti-tumor activity can be structurally modified to develop new anti-cancer drugs (Banik et al., 2022). Calycosin and formononetin with cardiovascular-protective effects can be further studied for drug or health-products development.

#### 3.5.2 Phenolic acids

A comprehensive analysis identified 280 phenolic acids in *E. japonica*, with LR exhibiting the highest accumulation, followed by S, MR, L, Inf, and Fr (**Supplementary Figure S3**). Comparative analyses revealed more up-regulated phenolic acids in LR, S, and Inf than in L, MR, and Fr, indicating higher concentrations in the former (**Supplementary Table S8**, **Supplementary Figure S13**). Among these, 21 were key active ingredients, and 12 were active pharmaceutical ingredients (**Supplementary Table S2**, **S9**). Four of these—grandidentatin, salidroside, syringin, and usnic acid—show resistance to nine major diseases (**Supplementary Table S2**). Salidroside, for instance, has been shown to reduce oxidative substances such as reactive oxygen species and malondialdehyde, thereby delaying the progression of aging-related diseases (Campisi et al., 2019). Though not meeting the criteria for key active ingredients, gallic and ferulic acids were predicted to resist all nine diseases (**Supplementary Table S2**). Gallic acid is known for its antioxidant, anti-inflammatory, antineoplastic activities, and therapeutic activities against gastrointestinal, neuropsychological, metabolic, and cardiovascular disorders (Kahkeshani et al., 2019). Ferulic acid is effective in treating diabetes, angina pectoris, heart stroke, coronary heart disease, and cardiovascular diseases (Liu et al., 2003; Guo et al., 2008). Of the six highlighted phenolic acids, except gallic acid (high in LR) and usnic acids (high in L), the other four were most abundant in Inf (**Supplementary Figure S25**). Given their significant antioxidant properties, the high levels of phenolic acids in *E. japonica*, especially in Inf, LR, and S, suggest its potential for improving human health.

#### 3.5.3 Amino acids and derivatives

Over 200 amino acids and derivatives were found in each tissue of *E. japonica*, representing over 90% of the total 220 (**Supplementary Figure S3**). Notably, Inf, LR, and Fr had higher concentrations (**Supplementary Table S8**, **Supplementary Figure S14**). None were key active ingredients, but 19 were active pharmaceutical ingredients. Six were predicted to correspond to nine major diseases: L-valine, 5-hydroxy-L-tryptophan, L-tryptophan, L-histidine, 5-hydroxy-DL-tryptophan, and N-methylphenylalanine (**Supplementary Table S2**). L-valine and L-histidine were more prevalent in MR and Fr, respectively, while 5-hydroxy-DL-tryptophan and N-methylphenylalanine were particularly abundant in Inf (**Supplementary Figure S26**).

#### 3.5.4 Lipids

In *E. japonica*, 205 lipids were detected across tissues, with LR containing the most (197) and Fr the least (168) (**Supplementary Figure S3**). Comparative analyses revealed more up-regulated lipids in Inf and LR, suggesting higher concentrations in these tissues (**Supplementary Table S8**; **Supplementary Figure S15**). Out of the total lipids, 25 were key active ingredients, and 16 were active pharmaceutical ingredients (**Supplementary Tables S2**, **S9**). Among these, 13 lipids were predicted to protect against nine major diseases, including nine most highly accumulated in Inf (9-hydroxy-10,12,15-octadecatrienoic acid, ethyl linoleate, linoleic acid, octadeca-9,12,15-trienoic acid, pinolenic acid, punicic acid, α-linolenic acid, and γ-linolenic acid, ricinoleic acid), two in LR (arachidonic acid, methyl linolenate), one in MR (elaidic acid) and one in Fr (stearic acid) (**Supplementary Figure S27**). Ricinoleic acid, linoleic acid, elaidic acid and stearic acid have also accumulated considerable amounts in other tissues (**Supplementary Figure S27**). Additionally, arachidonic acid and ethyl linoleate met criteria for potential drug candidates, with arachidonic acid playing a crucial role in cardiometabolic diseases (Hu et al., 2024), indicating its therapeutic potential in cardiovascular health. The higher relative content of these lipids in Inf and LR may enhance their value in protecting human health.

#### 3.5.5 Alkaloids

Among the 186 alkaloid metabolites detected in *E. japonica*, LR had the highest number of alkaloids (163), as well as richest accumulated alkaloids (53, 28.49%) (**Supplementary Figure S5F**). Differential expression and heatmap cluster analysis indicated a higher relative content of alkaloids in LR, Inf and L (**Supplementary Table S8**; **Supplementary Figure S16**). Nine alkaloids were core key active ingredients: lauroscholtzine, N-cis-feruloyltyramine, tetrahydroprotopapaverine, p-coumaroyltyramine, sophocarpine, sparteine, β-isosparteine, isomatrine, and moupinamide. Notably, the first four were core key active pharmaceutical ingredients, predicted to address nine major diseases, and significantly accumulated in LR (**Supplementary Table S2**; **Supplementary Figure S28**). Sophocarpine was also suggested to have anti-nociceptive, anti-inflammatory, neuroprotective, anti-tumor, and immune regulatory functions (Zhou et al., 2022), with higher relative content found in Fr and Inf. Additionally, sparteine and β-isosparteine were found to be significantly accumulated in S and were predicted to have anti-Alzheimer’s and analgesic effects, while isomatrine was notably present in Fr, L and Inf, and moupinamide was concentrated in LR.

#### 3.5.6 Organic acids

Of the 121 organic acids identified in *E. japonica*, the detectable quantities in each tissue ranged from 109 to 118 (**Supplementary Figure S3**). A high relative abundance of organic acids was also observed in Inf, Fr, and L, while MR and S exhibited lower levels (**Supplementary Table S8**; **Supplementary Figure S17**). Among the 41 organic acids recognized as components of traditional Chinese medicines in TCMSP, only two—anacardic acid and tianshic acid—were identified as key active ingredients (**Supplementary Table S2**). Anacardic acid, predicted to possess analgesic and anti-inflammatory properties, was found to be highly concentrated in Fr, whereas tianshic acid was relatively more abundant in Inf (**Supplementary Figure S29**).

#### 3.5.7 Lignans and coumarins

In MR, all lignans and coumarins identified in *E. japonica* were detected, totaling 103, while Fr detected the fewest, with only 76 (**Supplementary Figure S2**). Notably, LR contained the largest number of the richest accumulated lignans and coumarins, totaling 33 (**Supplementary Figure S5H**). Moreover, LR, S, and L also contained relatively high levels of lignans and coumarins (**Supplementary Table S8**; **Supplementary Figure S18**). A total of 20 lignans and coumarins were identified as key active ingredients, among which 10 were further categorized as core key active ingredients with potential for drug development. This subset included six lignans (acanthoside B, diphyllin, fargesin, glycyrin, picraquassioside C, and sesamin) and four coumarins (coumestrol, dalbergin, fraxin, and stevenin) (**Supplementary Table S2**). Furthermore, diphyllin, glycyrin, sesamin, dalbergin, and stevenin were predicted to be effective against nine major diseases, while coumestrol and fraxin were found to confer resistance to eight major diseases, excluding cardiovascular disease (**Supplementary Table S2**). The pharmacological properties of these lignans and coumarins have been continuously validated; for example, sesamin is known for its antioxidative, anti-cancerogenic, anti-inflammatory, anti-proliferative, anti-hypertensive, anti-melanogenesis, and promoting effect in treating osteoporosis (Yang et al., 2022; Dossou et al., 2023), highlighting its potential for various therapeutic applications. Coumestrol has also demonstrated anti-tumor activity by facilitating apoptosis in colorectal cancer cells (Geng et al., 2024). Here, considerable accumulation of sesamin was detected in all six tissues of *E. japonica*. Additionally, fargesin, picraquassioside C, acanthoside B, and coumestrol exhibited relatively high levels in MR, LR, and S. Fraxin and dalbergin were notably concentrated in LR, while stevenin was found in both LR and Fr, and glycyrin and diphyllin were primarily present in S and L, respectively (**Supplementary Figure S30**). These findings suggest that lignans and coumarins in various tissues of *E. japonica* significantly contribute to its pharmacological efficacy.

#### 3.5.8 Nucleotides and derivatives

A total of 72 nucleotides and derivatives were detected in *E. japonica*, with detectable quantities in each tissue ranging from 63 to 71 (**Supplementary Figure S3**). Inf and LR had higher levels of these compounds (**Supplementary Table S8**; **Supplementary Figure S19**). Six were identified as key active ingredients: 2’-deoxyadenosine, adenosine, cordycepin, guanosine, uridine 5’-diphosphate, and uridine 5’-monophosphate (**Supplementary Tables S2**, **S9**). Their concentrations across various tissues were presented in **Supplementary Figure S31**. Additionally, 2’-deoxyadenosine and cordycepin were predicted to be associated with nine major diseases (**Supplementary Table S2**). Existing studies have also confirmed that cordycepin, recognized as a potential anti-cancer/tumor agent, exhibits complementary therapeutic activities in promoting apoptosis, anti-proliferation, and anti-metastasis in cancer cells (Yoon et al., 2018; Schwenzer et al., 2021).

#### 3.5.9 Terpenoids

The highest number of terpenoids was detected in LR (67), followed by MR (65) (**Supplementary Figure S3**). Differential metabolite analysis revealed that the relative contents of many terpenoids in LR were significantly higher than those in other tissues (**Supplementary Table S8**; **Supplementary Figure S20**). A total of 21 terpenoids were identified as key active ingredients, comprising 16 triterpenes, four diterpenoids, and one triterpene saponin (**Supplementary Table S9**). Among these, two triterpenes (asiatic acid, and mangiferonic acid) and three diterpenoids (gibberellin A3, isopimaric acid, and kaurenoic acid) met the criteria of core key active ingredients (**Supplementary Table S2**; **Supplementary Figure S32**). Asiatic acid, despite no TCMSP disease annotations, has shown anti-inflammatory, antioxidant, anti-fibrosis, and anti-tumor properties in diverse disease models (Lv et al., 2017; Chung et al., 2021), and was most abundant in LR, indicating the tissue’s potential health benefits.

#### 3.5.10 Quinones

A total of 22 quinones were detected in *E. japonica*, with approximately 21 detected in all tissues except for Inf, with only 13 identified in Inf (**Supplementary Figure S2**). LR and Fr contained more up-regulated quinones than down-regulated ones compared to other tissues, indicating higher overall quinone levels in LR and Fr (**Supplementary Table S8**; **Supplementary Figure S21**). Seven quinones were identified as key active ingredients, including three highly accumulated quinones in LR (1,2,4-trihydroxyanthraquinone, physcion, and rheic acid), two highly accumulated quinones in Fr (emodin-8-O-glucoside, and helminthosporin), one quinone shared between LR and Fr (rubiadin-1-methyl ether), and one quinone (embelin) that was commonly found in all tissues (**Supplementary Table S2**; **Supplementary Figure S33**). Furthermore, embelin, emodin-8-O-glucoside, and rheic acid were classified as core key active ingredients. Additionally, 1,2,4-trihydroxyanthraquinone, physcion, rheic acid, and embelin were annotated as active pharmaceutical ingredients associated with nine or eight major diseases (**Supplementary Table S2**). The higher accumulation of these quinones in LR and/or Fr suggested that they significantly contribute to the health benefits attributed to these tissues.

#### 3.5.11 Tannins

Tannins were predominantly detected in LR, where 14 out of 15 tannins were identified, with 10 exhibiting the highest relative content or accumulating exclusively in LR (**Supplementary Figures S3**, **S5L**, **S22**). Among the 15 tannins, only seven were annotated in TCMSP, of which four were annotated as key active ingredients: 3,3’-di-O-methylellagic acid 4’-glucoside, ellagic acid, procyanidin B1, and procyanidin B4 (**Supplementary Tables S2**, **S9**). Metabolite content analysis revealed that ellagic acid accumulated at comparable levels across all six tissues, while 3,3’-di-O-methylellagic acid 4’-glucoside mainly accumulated in high concentrations in S and L. In contrast, procyanidin B1 and procyanidin B4 were highly or exclusively accumulated in LR (**Supplementary Figure S34**).

#### 3.5.12 Others

In addition to the aforementioned metabolites, a total of 207 metabolites from eight other categories were detected in *E. japonica* (**Figure 2B**), with quantities ranging from 191 to 200 in each tissue (**Supplementary Figure S3**). Besides saccharides being most abundant in MR, other types of metabolites were also mainly accumulated in LR and Inf (**Supplementary Table S8**; **Supplementary Figures S23**, **S24**). Of the 64 metabolites annotated in TCMSP, only 12 were annotated as key active ingredients, while 25 were identified as active pharmaceutical ingredients, with eight metabolites co-annotated (**Supplementary Table S2**). The 12 key active ingredients included three chromones, three saccharides, three vitamins, two ketone compounds, and 3’-O-methylorobol (**Supplementary Table S2**; **Supplementary Figure S35**). Among these metabolites, capillarisin, vitamin K1, and 3’-O-methylorobol met the criteria of OB ≥ 30% and DL ≥ 0.18, and were associated with nine or eight major diseases, indicating their potential for new drug development (**Supplementary Table S2**).

In summary, *E. japonica*’s tissues showed distinct metabolite profiles. LR had higher accumulations of most metabolite classes, especially flavonoids, phenolic acids, alkaloids, lignans and coumarins, terpenoids, quinones, and tannins. Inf was rich in many metabolite classes except lignans and coumarins, quinones, and tannins. In other tissues, the accumulation of certain metabolites was relatively abundant. For instance, the content of flavonoids, alkaloids and organic acids was relatively high in L; phenolic acids, lignans and coumarins were richly accumulated in S; organic acids and quinones were also abundant in Fr. However, except for saccharides that predominantly accumulated in MR, the content of other metabolites in MR was comparatively lower than that in other tissues. These findings indicate that for *E. japonica*, with its roots used as folk medicine, the medicinal efficacy likely derives mainly from LR, as LR generally contains a higher content of pharmacologically active components than MR.

Overall, the diverse chemical composition across *E. japonica* tissues points to its significant pharmacological potential. Notably, LR and Inf demonstrate the greatest application potential due to their rich profiles of bioactive compounds, suggesting they could serve as key sources for developing treatments for various diseases, particularly for treating chronic and multifactorial diseases. This study underscores the pharmacological promise of *E. japonica* in medicinal applications, warranting further investigation into these specific tissues for drug development.

### 3.6 KEGG annotation and enrichment analysis of DEMs

To explore *E. japonica*’s metabolic pathways across different tissues, the KEGG and MetMap databases were used to annotate and analyze 2045 DEMs. A total of 416 DEMs were annotated in KEGG, 276 in MetMap, with 43 co-annotated (**Supplementary Table S1**). These DEMs were assigned into 101 KEGG metabolic pathways and 16 MetMap metabolic pathways (**Supplementary Figure S36**). Subsequent enrichment analysis and mapping of DEMs across 15 comparisons among the six tissues revealed involvement in various metabolic pathways, with 101–113 pathways in each comparison. Notably, 153 DEMs were mapped to the biosynthesis of secondary metabolites. Furthermore, the number of DEMs associated with the biosynthesis of cofactors, biosynthesis of amino acids, ABC transporters, biosynthesis of isoflavones aglycones I, flavonoid biosynthesis, and isoflavonoid biosynthesis was particularly large, highlighting their significance in *E. japonica*’s metabolism (**Supplementary Figure S36**).

#### 3.6.1 KEGG enrichment analysis of DEMs

**Figure 7:**
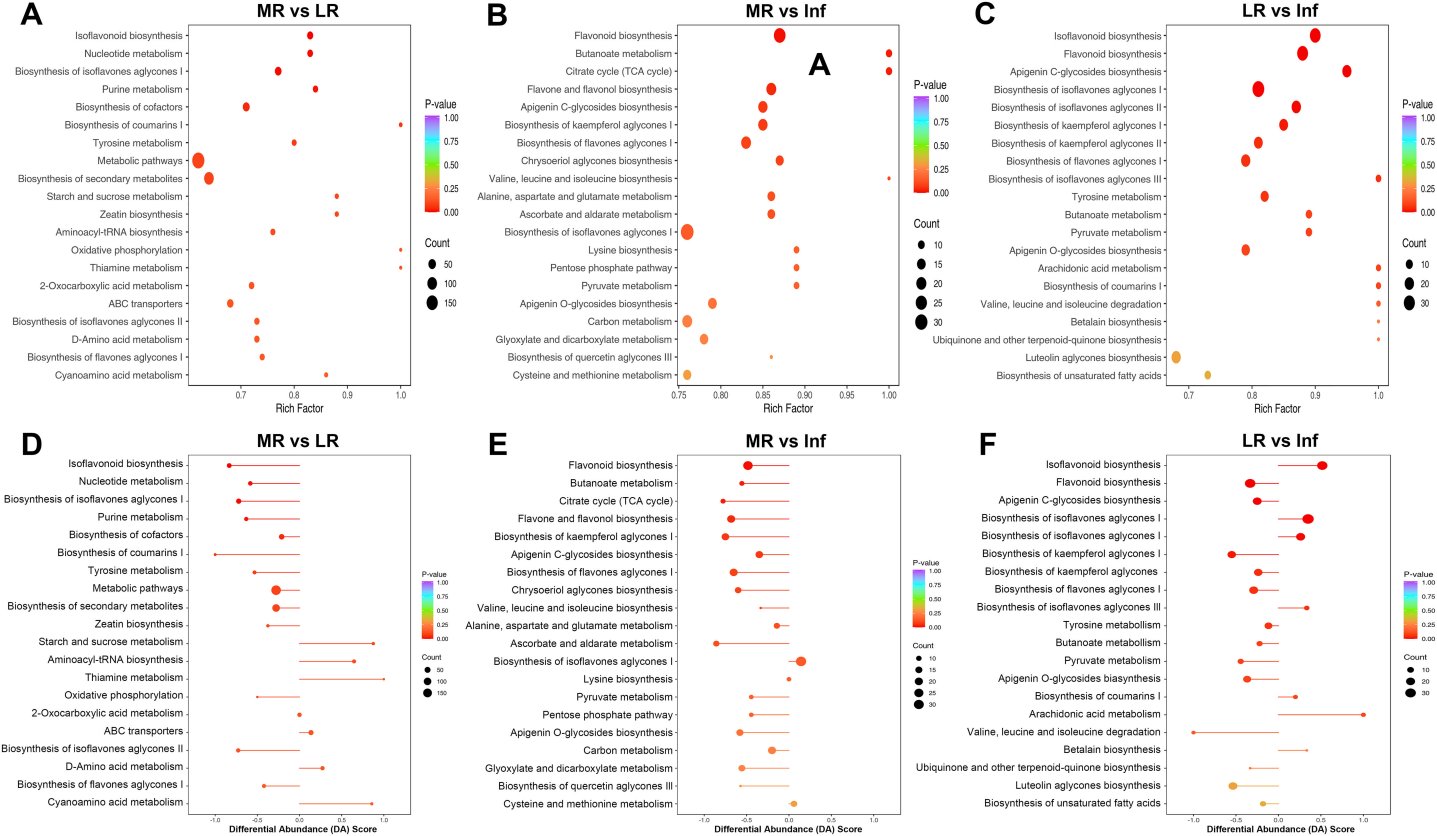
KEGG pathway enrichment analysis of differentially expressed metabolites (DEMs) of each pairwise comparison among MR, LR and Inf. KEGG annotations and enrichment of DEMs of MR vs. LR **(A)** MR vs. Inf **(B)** and LR vs. Inf **(C)**. Differential abundance (DA) score of DEMs of MR vs. LR **(D)** MR vs. Inf **(E)** and LR vs. Inf **(F)**. MR, LR, and Inf represent main-roots, lateral-roots, and inflorescence, respectively. The top 20 most significantly enriched pathways identified through KEGG enrichment analysis were presented.

KEGG pathway enrichment analysis was conducted based on the characteristics of the DEMs. In KEGG enrichment analysis, the rich factor reflects the degree of enrichment; a value closer to 1 indicates a higher proportion of annotated metabolites within a corresponding pathway that are classified as DEMs. The top 20 pathways identified via KEGG enrichment analysis were shown in **Supplementary Table S10**, **Figure 7** and **Supplementary Figures S37–39**.

Among the top 20 pathways across the 15 comparisons, a total of 88 pathways were identified, with 32 pathways significantly enriched (*p* < 0.05) in at least one comparison (**Supplementary Table S10**; **Supplementary Figure S37**). Notably, six pathways—biosynthesis of isoflavones aglycones I, II, and III, and biosynthesis of flavones aglycones I, II, and III—ranked among the top 20 pathways in most comparisons and were significantly enriched in several cases. Additionally, ten pathways, including starch and sucrose metabolism, flavonoid biosynthesis, isoflavonoid biosynthesis, galactose metabolism, pentose and glucuronate interconversions, biosynthesis of cofactors, ascorbate and aldarate metabolism, biosynthesis of kaempferol aglycones I, carbon fixation in photosynthetic organisms, and nucleotide metabolism, were significantly enriched in at least two comparisons (**Supplementary Table S10**; **Supplementary Figure S37**). Overall, the pathways mentioned above are likely responsible for the significant differences in chemical composition observed among the six tissues of *E. japonica*.

#### 3.6.2 Overall changes in KEGG metabolic pathway

**Figure 8:**
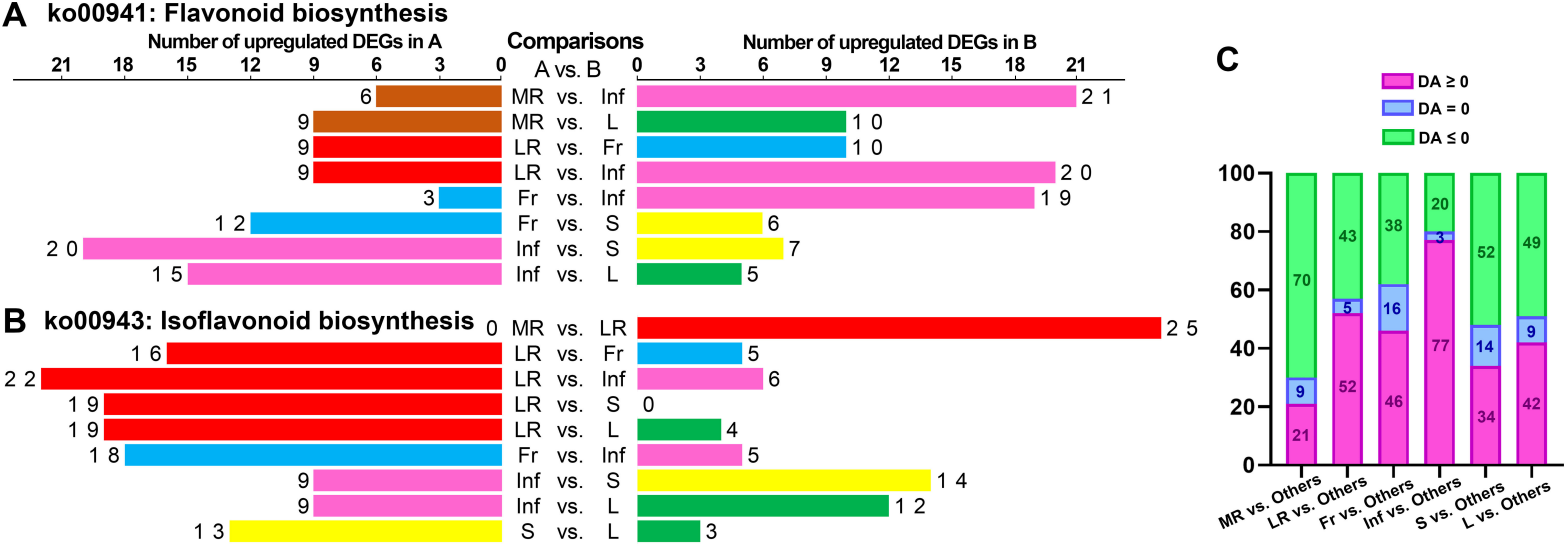
Statistical analysis of the number of up-regulated metabolites enriched in specific KEGG pathways in each tissue of each pairwise comparison. The number of up-regulated metabolites enriched in “flavonoid biosynthesis” (ko00941) **(A)** and “isoflavonoid biosynthesis” (ko00943) **(B)** in each tissue of each pairwise comparison. **(C)** Statistical analysis of differential abundance (DA) scores for the top 20 KEGG pathways in pairwise comparison between each tissue and five other tissues.

The DA score is a pivotal metric for metabolite changes within metabolic pathways, with values closer to 1 indicating more up-regulated DEMs and -1 indicating more down-regulated DEMs. The top 20 pathways, ranked by *p*-values, were detailed in **Supplementary Table S10**, yielding 300 records across 88 pathways from 15 comparisons (**Supplementary Figure S37**). Notably, the “biosynthesis of flavones aglycones I” and “biosynthesis of isoflavones aglycones I” pathways were most prominent, featuring in 12 of the 15 comparisons. Other notable pathways included “biosynthesis of isoflavones aglycones II,” “biosynthesis of flavones aglycones II,” “biosynthesis of flavones aglycones III,” “isoflavonoid biosynthesis,” and “flavonoid biosynthesis”, which appeared in at least eight comparisons. This underscores the significant divergence in flavonoid and isoflavonoid biosynthesis across tissues. Up-regulated DEM distribution revealed that the expression of “flavonoid biosynthesis” pathway was most active in Inf, while “isoflavonoid biosynthesis” was most abundant in LR (**Figure 8**). In similar studies of *Astragalus membranaceus* and *Sophora flavescens*, flavonoids have also been confirmed as their main bioactive components, and these components are highly accumulated in the roots. The studies have also identified flavonoid and isoflavonoid biosynthesis as critical pathways, highlighting their conserved functions in medicinal legumes (Wu et al., 2020; Wei et al., 2021). Revealing flavonoid and isoflavonoid biosynthetic pathways and their regulation in medicinal legumes will provide a key theoretical basis for cultivating high-bioactivity plants in the future.

In the comparative analysis of each tissue against others, the top 20 pathways were selected, and DA scores for 100 pathways per tissue were statistically analyzed. Inf vs. MR/LR/Fr/S/L showed the most pathways (77) with DA scores above zero, indicating higher expression, followed by LR (52), Fr (46), L (42), S (34), and MR (21) (**Figure 8C**). These findings suggest that the expression of numerous pathways in Inf and LR was more abundant than in other tissues, whereas MR showed relatively fewer pathways expressed at higher levels compared to the other tissues.

In the MR vs. LR/Fr/Inf/S/L comparisons, only 6, 3, 2, 4, and 6 pathways exhibited DA scores greater than zero, respectively (**Figures 7D, E**; **Supplementary Figures S39A–C**). Additionally, only two pathways—”starch and sucrose metabolism” and “galactose metabolism”—in the MR vs. L, were significantly enriched with DA scores above zero, indicating relatively high expression of these pathways in MR. In contrast, in the LR vs. MR/Fr/Inf/S/L comparisons, more pathways had DA scores greater than zero (**Figures 7D, F**; **Supplementary Figures S39D–F**). Besides those involved in isoflavonoid biosynthesis, metabolites related to “arachidonic acid metabolism,” “biosynthesis of coumarins I,” “biosynthesis of various alkaloids,” “alpha-linolenic acid metabolism,” and “benzoxazinoid biosynthesis” were also relatively abundant in LR. Inf vs. MR/LR/Fr/S/L comparisons highlighted a greater number of pathways with higher DA scores (**Figures 7B, C**; **Supplementary Figures S39G, J, K**), indicating that many pathways were expressed more abundantly in Inf than in other tissues. In addition to pathways associated with flavonoid biosynthesis, pathways with high expression in Inf also included “butanoate metabolism,” “pyruvate metabolism,” “apigenin O-glycosides biosynthesis,” “apigenin C-glycosides biosynthesis,” “alanine, aspartate and glutamate metabolism,” and “biosynthesis of kaempferol aglycones I”. In the comparisons of Fr vs. MR/LR/Inf/S/L, S vs. MR/LR/Fr/Inf/L, and L vs. MR/LR/Fr/Inf/L, the number of pathways with high DA scores varied (**Supplementary Figure S39**). The expression levels of several pathways in these tissues were also noteworthy. For example, the expression of “luteolin aglycones biosynthesis” in Fr was more abundant than in MR/LR/S, while “pentose and glucuronate interconversions” was expressed at higher levels in L compared to MR/LR/Inf/S. Conversely, the expression of “starch and sucrose metabolism” in L was significantly lower than in MR/Fr/Inf/S.

## 4 Conclusion

In this study, we conducted a UPLC-ESI-Q TRAP-MS/MS-based widely targeted metabolomics analysis to systematically identify metabolites across six tissues of *E. japonica* for the first time. Our findings provided firsthand comprehensive information on the composition and abundance of metabolites, revealing that flavonoids were the most abundant, followed by phenolic acids, amino acids and derivatives, lipids, and alkaloids. Distinct metabolite profiles were observed among the different tissues. Notably, LR and Inf exhibited higher concentrations for most metabolite categories compared to other tissues. L, S, and Fr also showed elevated levels in several metabolite categories, while the MR contained lower levels except for saccharides. This work also provided the information of the key and iconic health-promoting compounds in *E. japonica*, identifying 305 metabolites as key active ingredients and 364 metabolites as active pharmaceutical ingredients, of which 206 were co-annotated. Given the high concentration of these active pharmaceutical ingredients, *E. japonica* shows great promise in pharmaceutical applications. For instance, extracts rich in these metabolites could potentially be developed into drugs for treating chronic inflammatory diseases, considering the anti-inflammatory properties often associated with flavonoids and phenolic acids. Additionally, they might be used in the development of nutraceuticals to improve overall health and boost the immune system. Moreover, the comparative analysis of LR and MR indicates that the therapeutic efficacy of *E. japonica* roots, commonly utilized in folk medicine, is primarily attributed to the lateral roots rather than the main roots. These observations underscore the importance of prioritizing the application of lateral roots in future therapeutic strategies involving *E. japonica*. Overall, these findings highlight the strongest application potential of LR and Inf for disease treatment, particularly for addressing chronic and multifactorial diseases due to their high concentrations of bioactive compounds. The notable accumulation of therapeutic metabolites in *E. japonica* indicates its potential as a valuable resource for novel treatments, warranting further investigation into its pharmacological properties and applications in drug development.

## Data Availability

The original contributions presented in the study are included in the article/**Supplementary Material**. Further inquiries can be directed to the corresponding authors.
