# Supplementary material for: Metabolomic profiles and health-promoting potential of Euchresta japonica tissues revealed by widely targeted metabolomics
Source: Front Plant Sci. 2025 May 1;16:1537273. doi: 10.3389/fpls.2025.1537273 (PMC12078262; doi:10.3389/fpls.2025.1537273)
Supplement: Supplementary file 11 [file DataSheet11.docx]

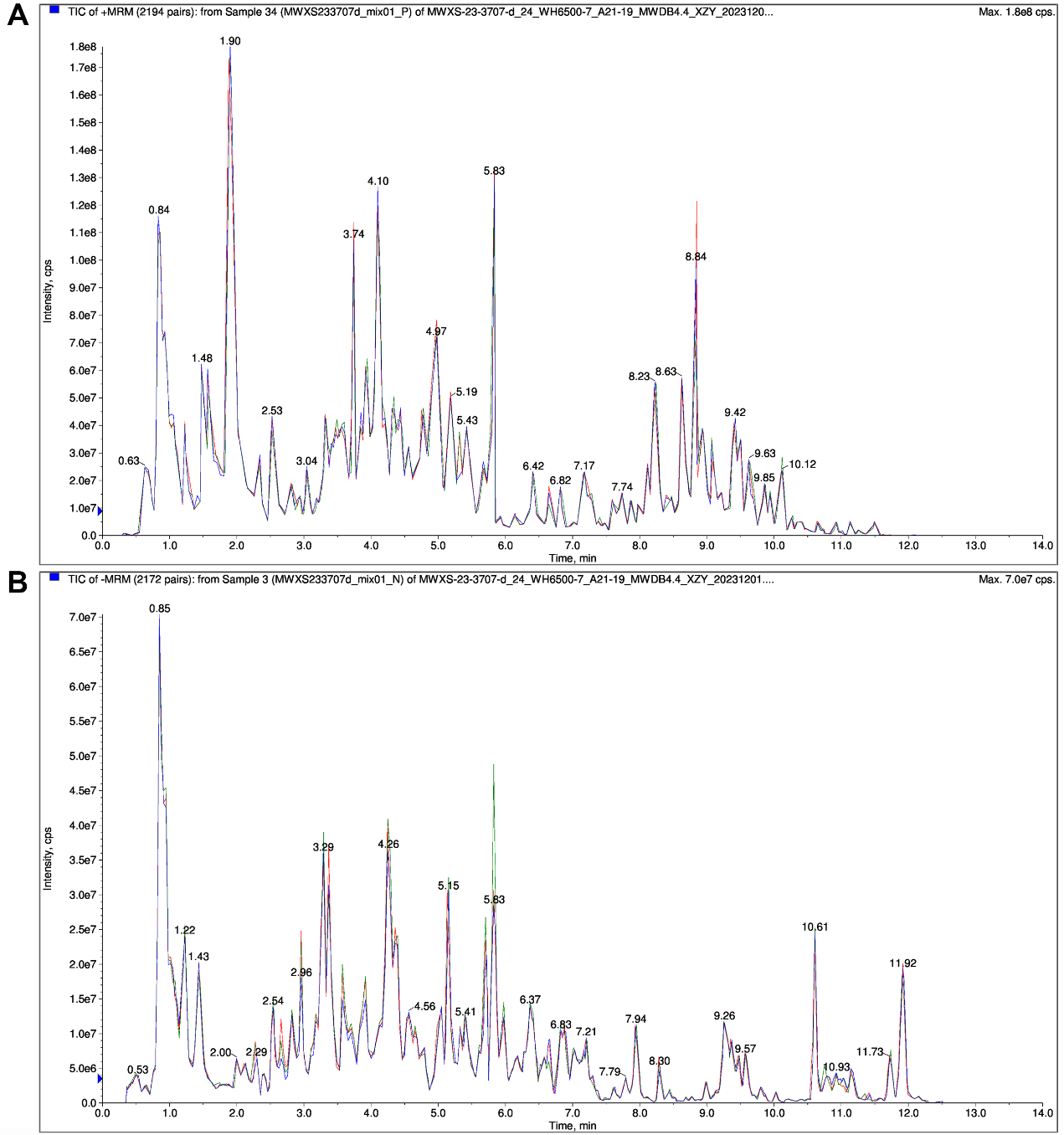


**Supplementary Figure 1.** Total ions current (TIC) overlapping map of QC sample mass spectrometry results in positive (A) and negative (B) ion mode.


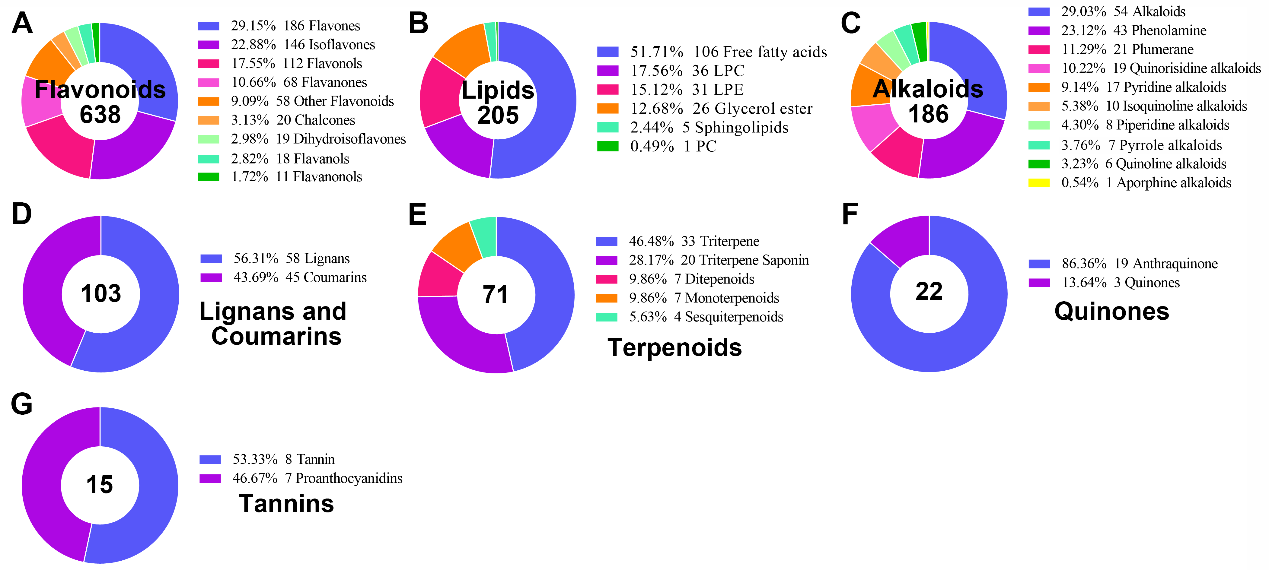


**Supplementary Figure 2.** The specific classification of each metabolite category and the number of metabolites identified in *Euchresta japonica*.


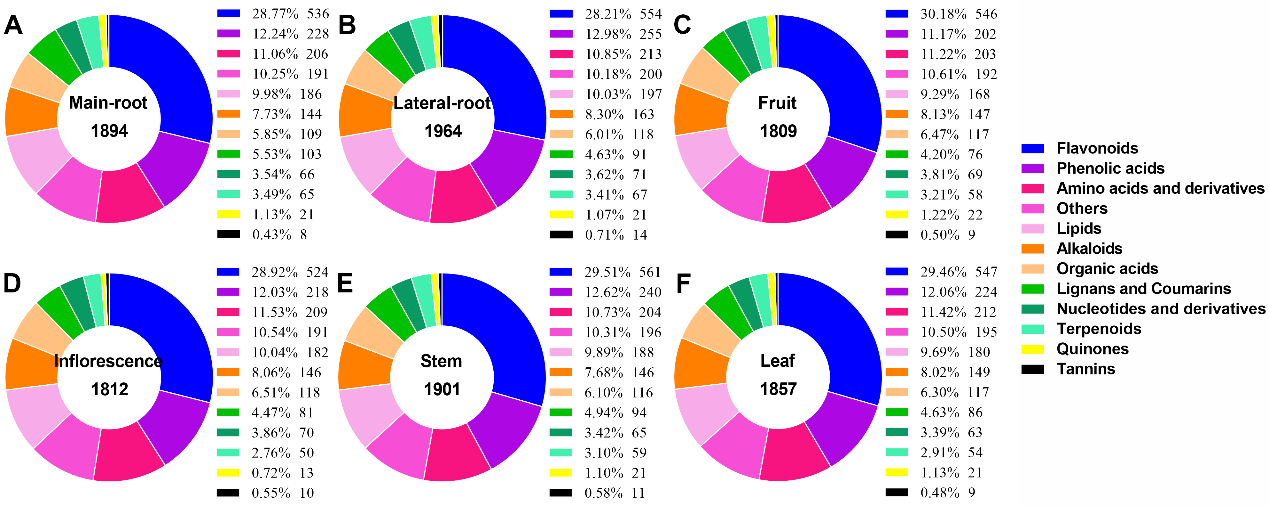


**Supplementary Figure 3.** Classification of metabolites identified in each of the six tissues of *Euchresta japonica*.


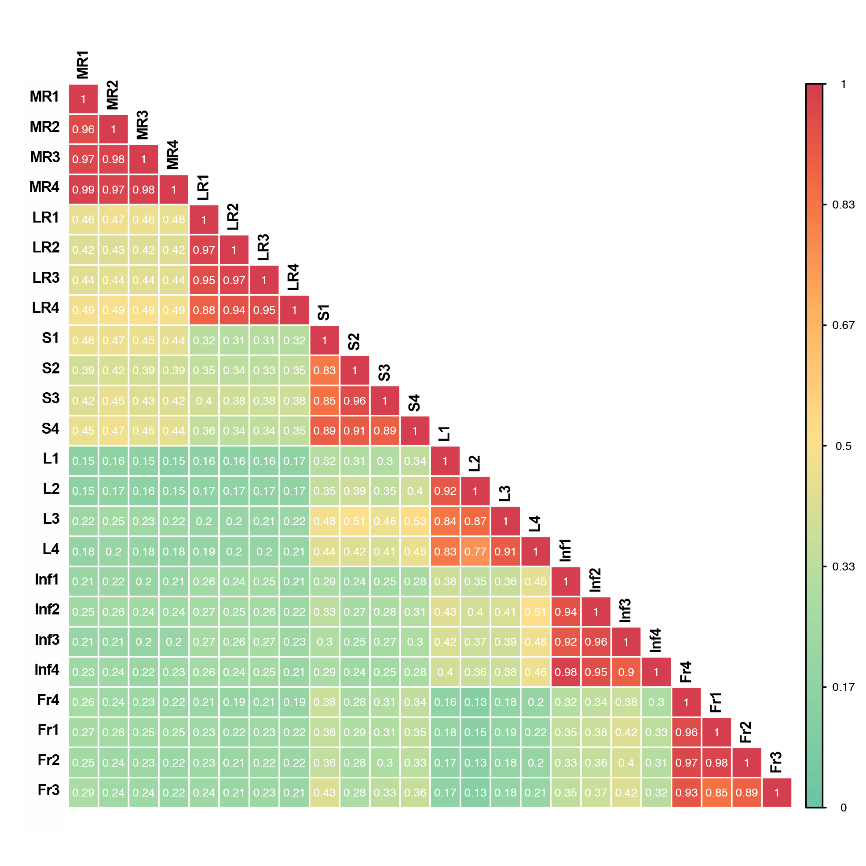


**Supplementary Figure 4.** Pearson correlation coefficients (PCC) of the samples.


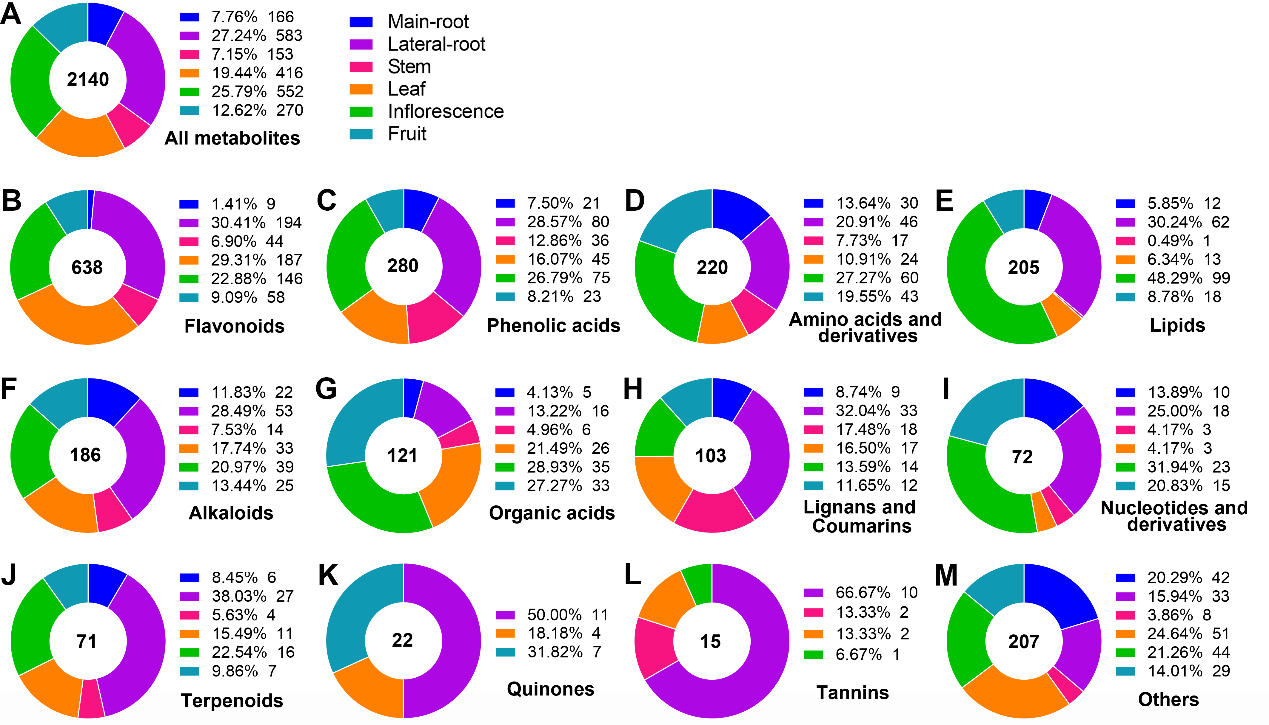


**Supplementary Figure 5.** Statistical analysis of the distribution of tissues with the richest accumulation of metabolites identified in *Euchresta japonica*.


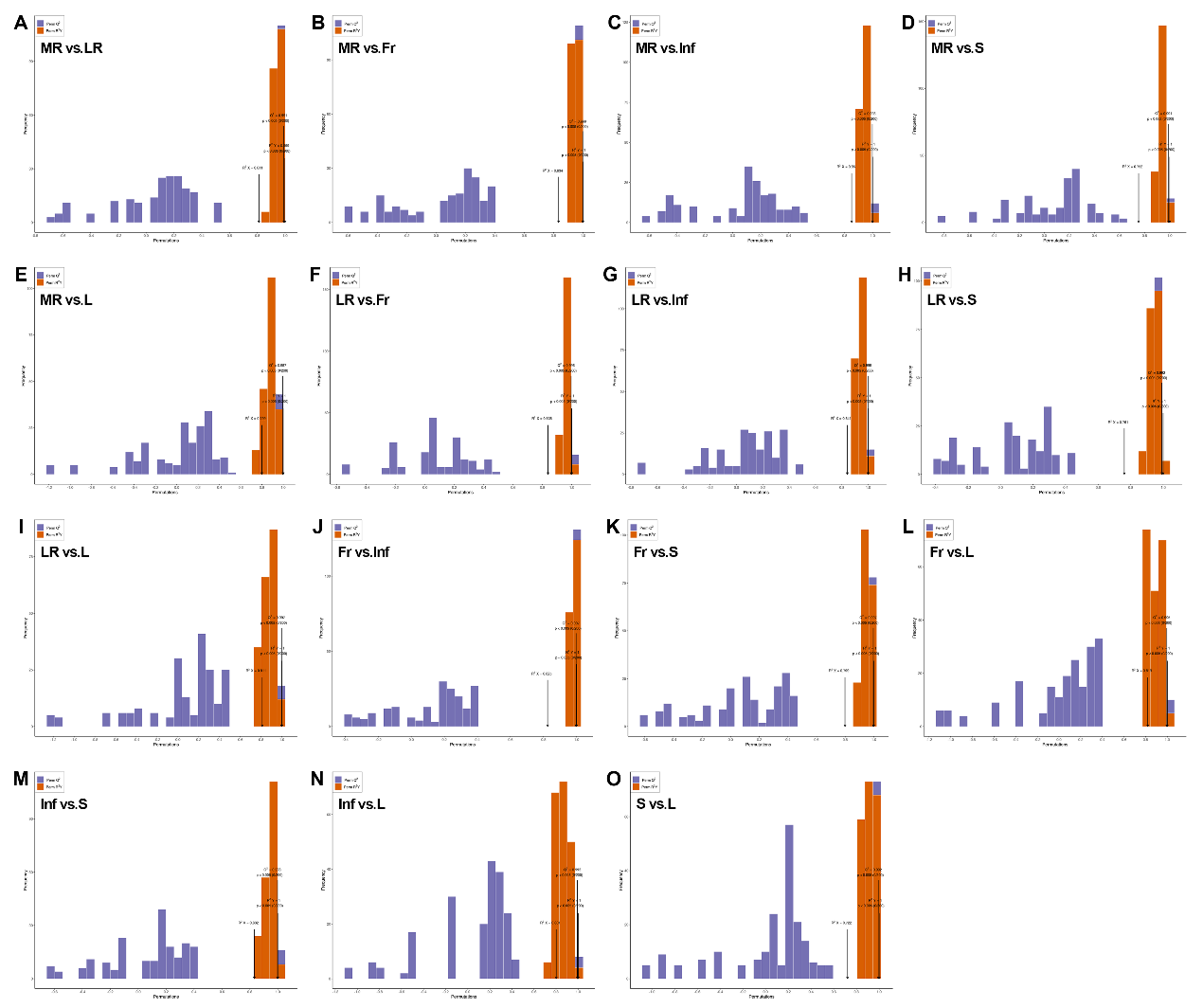


**Supplementary Figure 6.** OPLS-DA validation diagrams. In the diagram, orange represents the random grouping model R^2^Y, purple represents the random grouping model Q^2^, and the values represented by black arrows are the R^2^X, R^2^Y and Q^2^ values of the original model. (A-E), MR vs. LR/Fr/Inf/S/L; (F-I), LR vs. Fr/Inf/S/L; (J-L), Fr vs. Inf/S/S; (M-N), Inf vs. S/L; (O), S vs. L.


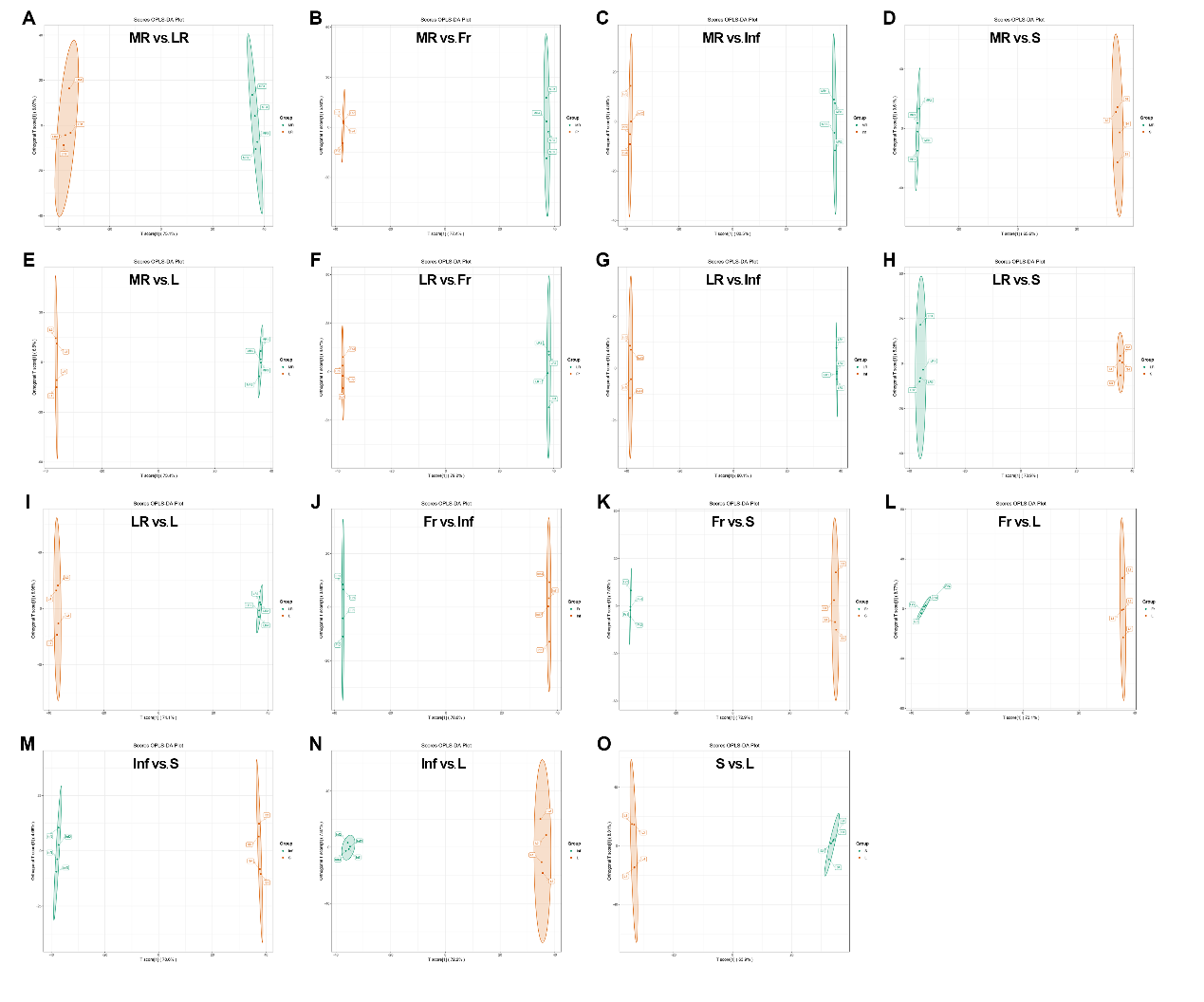


**Supplementary Figure 7.** Orthogonal projections to latent structures-discriminant analysis (OPLS-DA). Score scatter plots of the OPLS-DA model for MR vs. LR/Fr/Inf/S/L (A-E), LR vs. Fr/Inf/S/L (F-I), Fr vs. Inf/S/L (J-L), Inf vs. S/L (M-N), S vs. L(O).


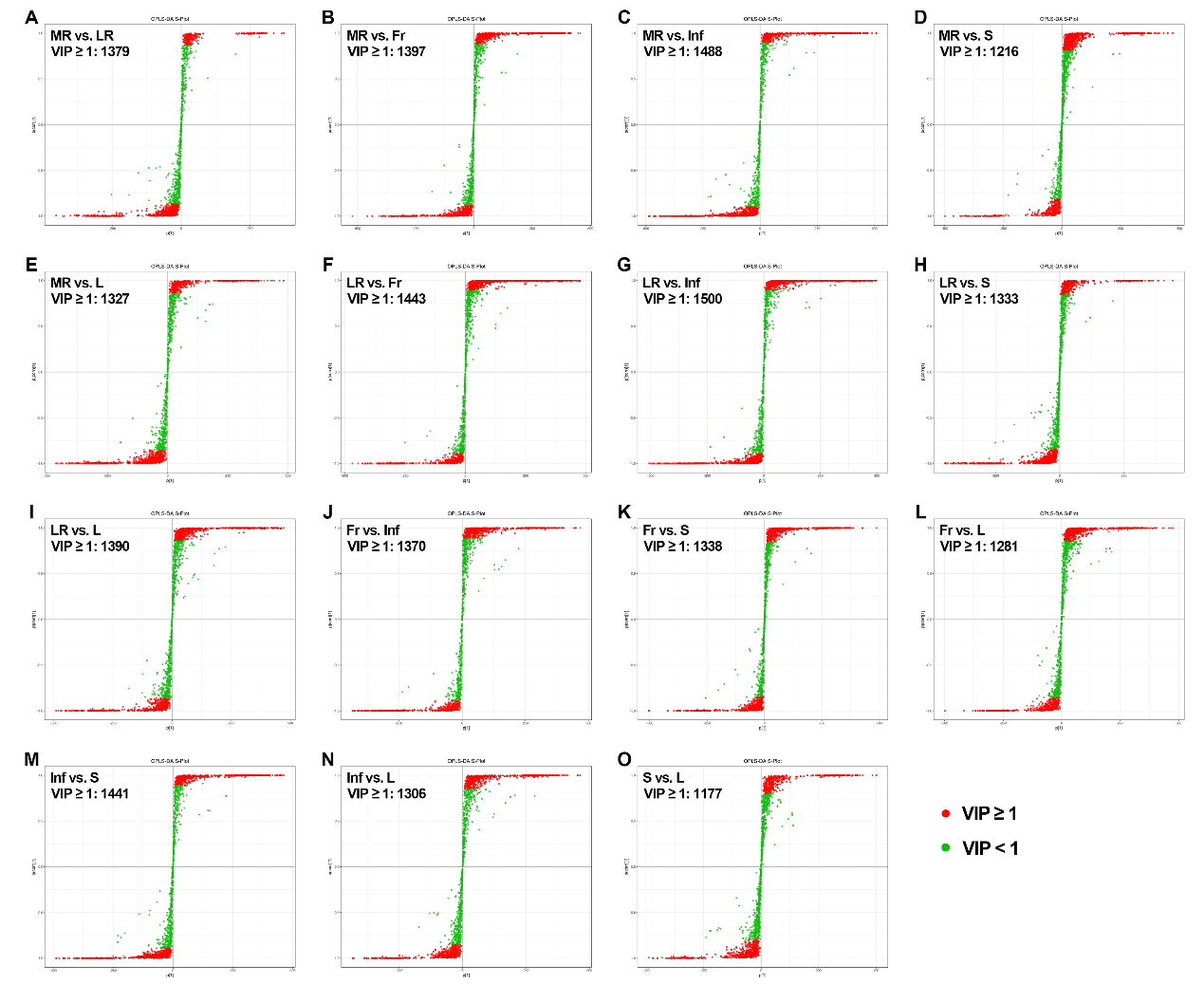


**Supplementary Figure 8.** S-plots of the OPLS-DA model for MR vs. LR/Fr/Inf/S/L (A-E), LR vs. Fr/Inf/S/L (F-I), Fr vs. Inf/S/L (J-L), Inf vs. S/L (M-N), S vs. L(O).


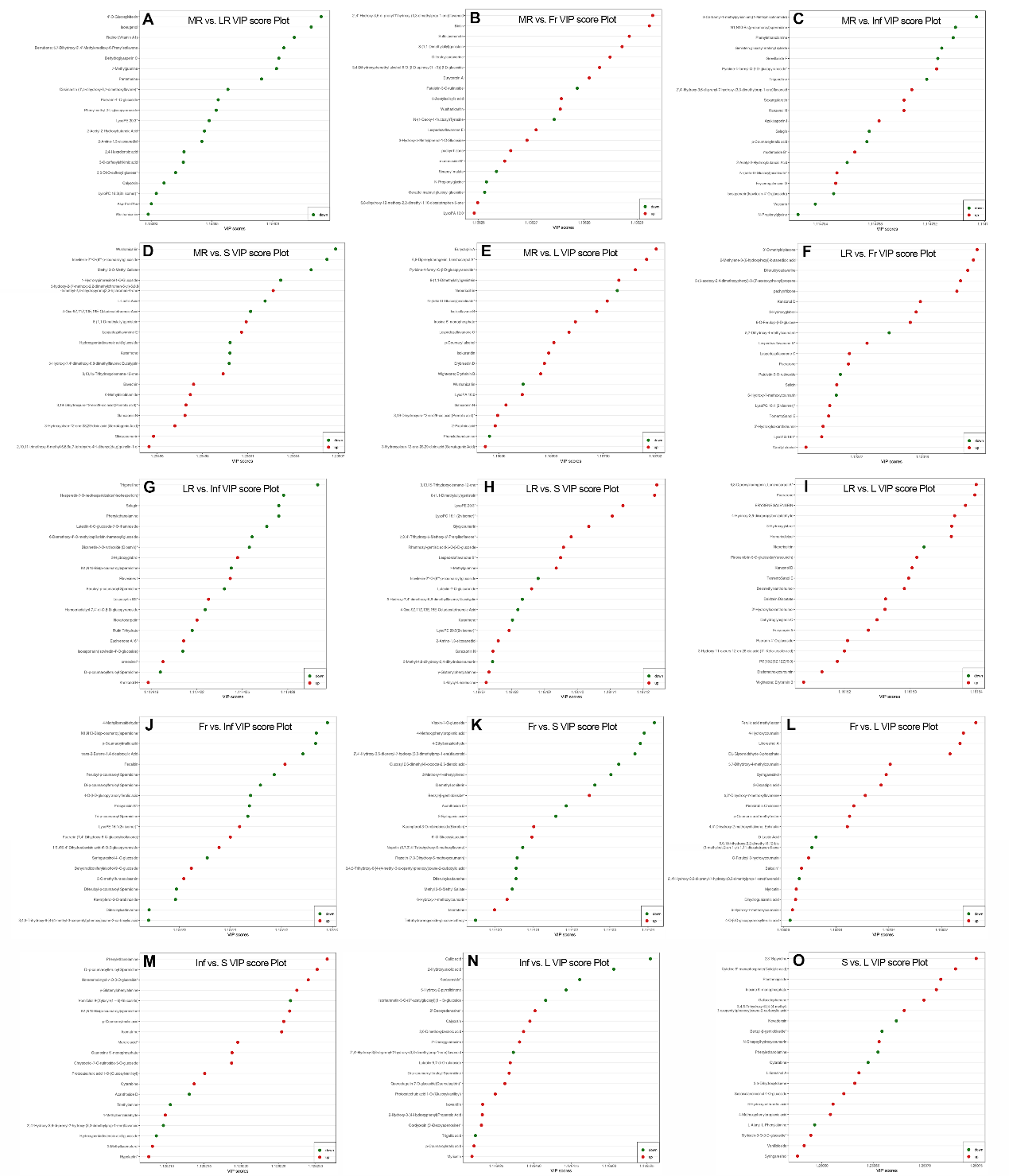


**Supplementary Figure 9.** Top 20 metabolites with highest VIP scores of MR vs. LR/Fr/Inf/S/L (A-E), LR vs. Fr/Inf/S/L (F-I), Fr vs. Inf/S/L (J-L), Inf vs. S/L (M-N), S vs. L(O).


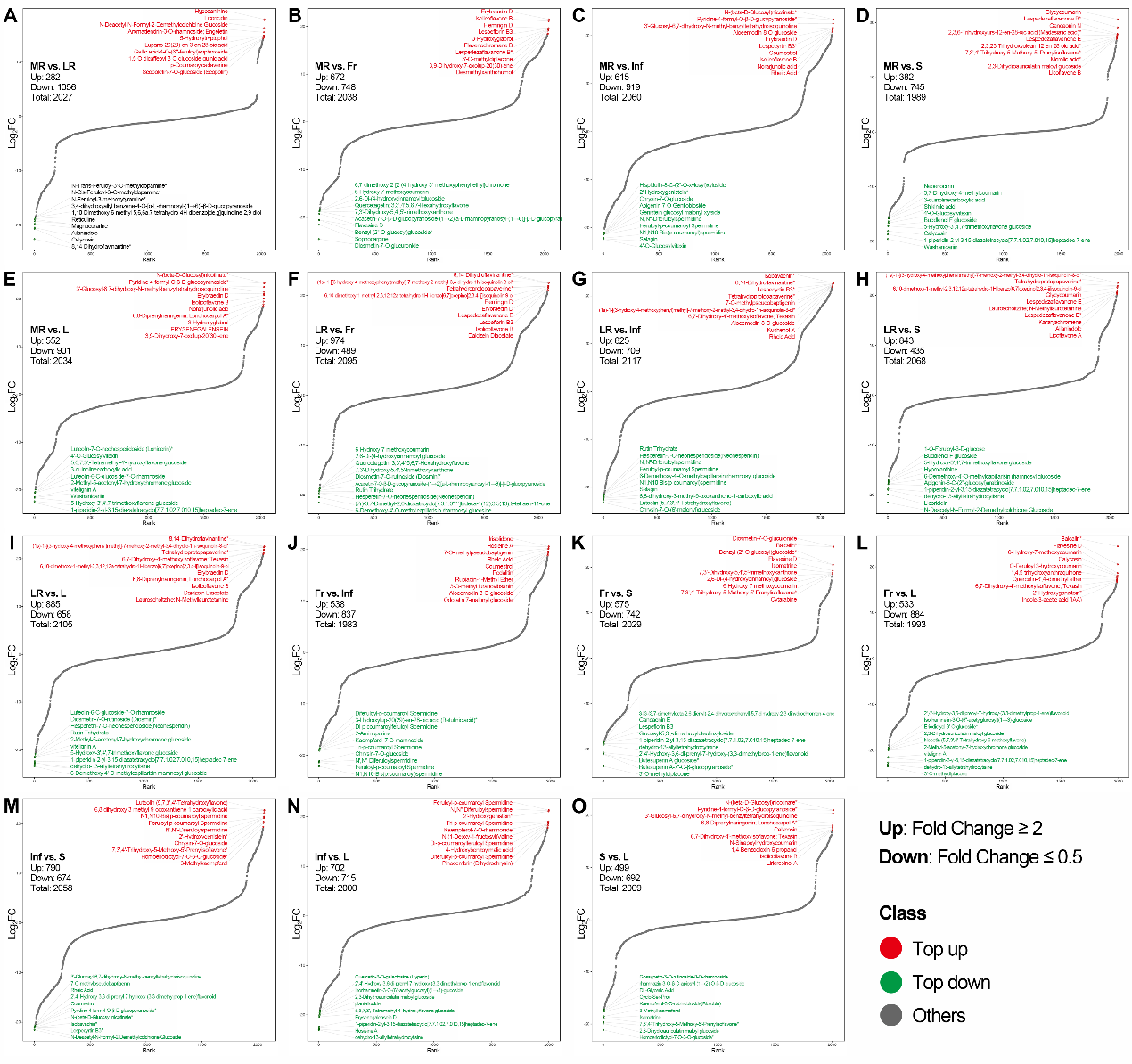


**Supplementary Figure 10.** Dynamic distribution diagram of metabolite content difference of each pairwise comparison of the six tissues of *Euchresta japonica*. (A-E), MR vs. LR/Fr/Inf/S/L; (F-I), LR vs. Fr/Inf/S/L; (J-L), Fr vs. Inf/S/S; (M-N), Inf vs. S/L; (O), S vs. L.


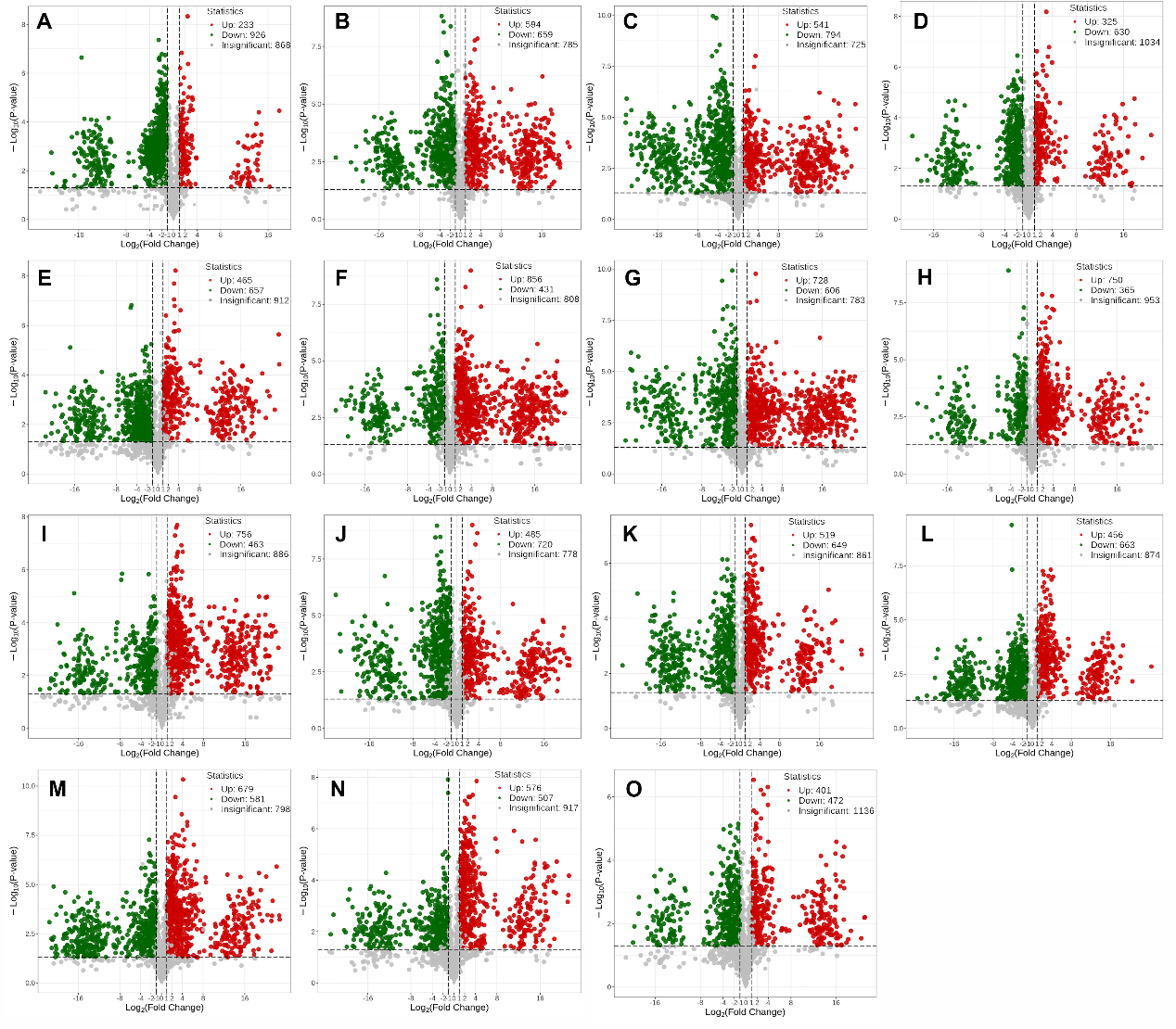


**Supplementary Figure 11.** Volcano plot of the differential metabolites of MR vs. LR/Fr/Inf/S/L (A-E), LR vs. Fr/Inf/S/L (F-I), Fr vs. Inf/S/L (J-L), Inf vs. S/L (M-N), S vs. L(O). The criteria set at VIP ≥ 1, FC ≥ 2 or ≤ 0.05. Red and green dots represent up-regulated and down-regulated differential metabolites, respectively. Gray dots represent non-differential metabolites.


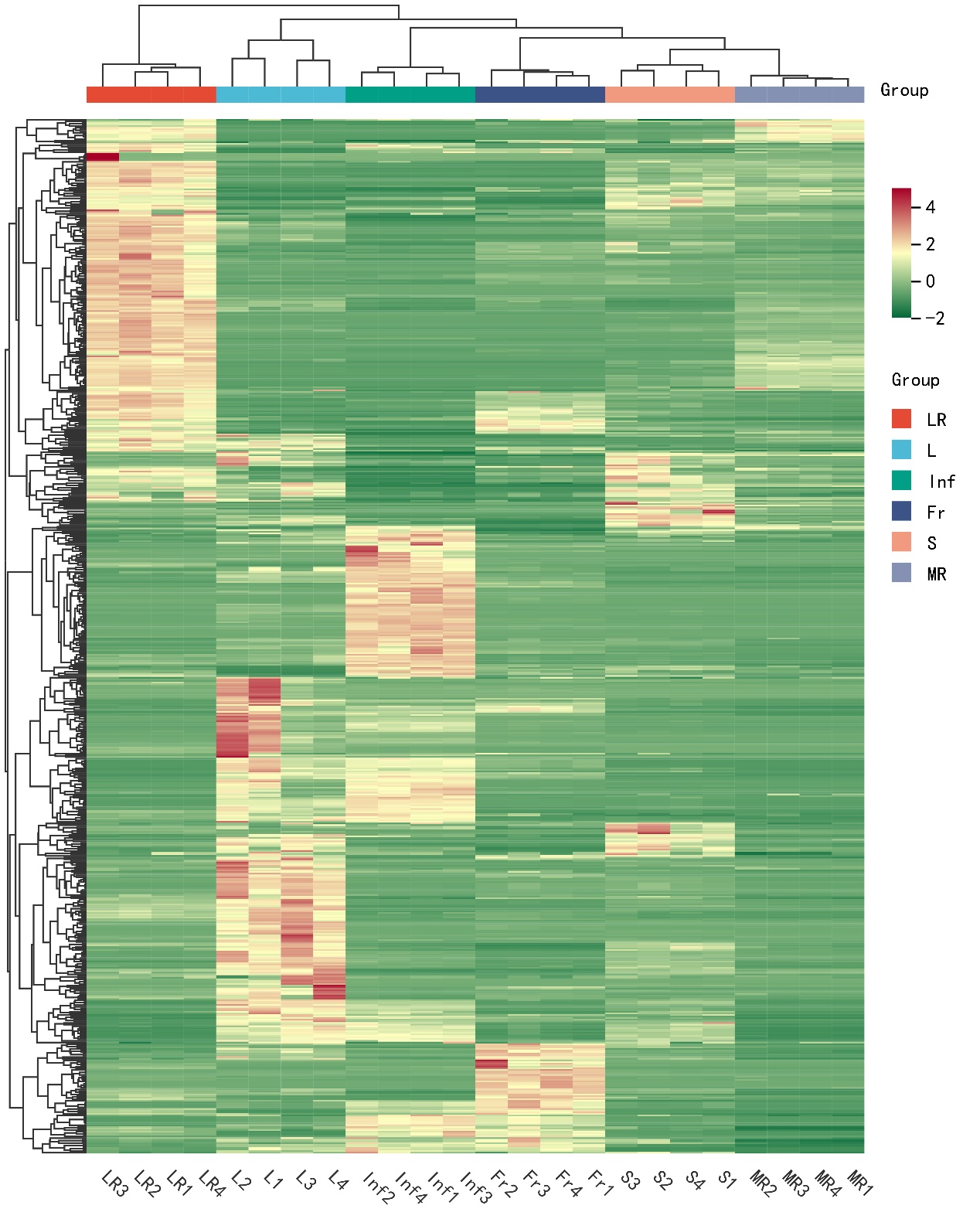


**Supplementary Figure 12.** Heatmap analysis of the levels of flavonoids.


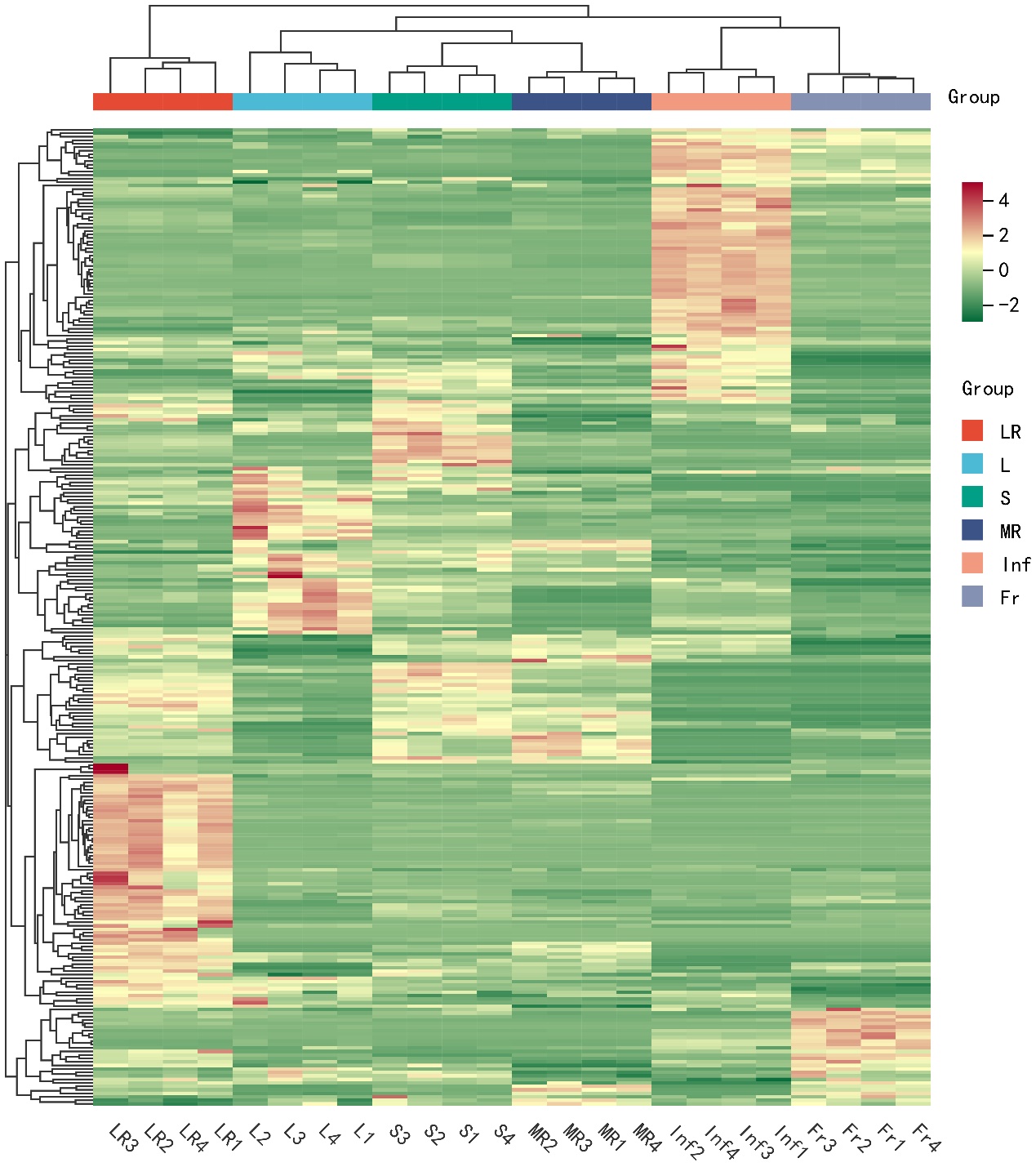


**Supplementary Figure 13.** Heatmap analysis of the levels of phenolic acids.


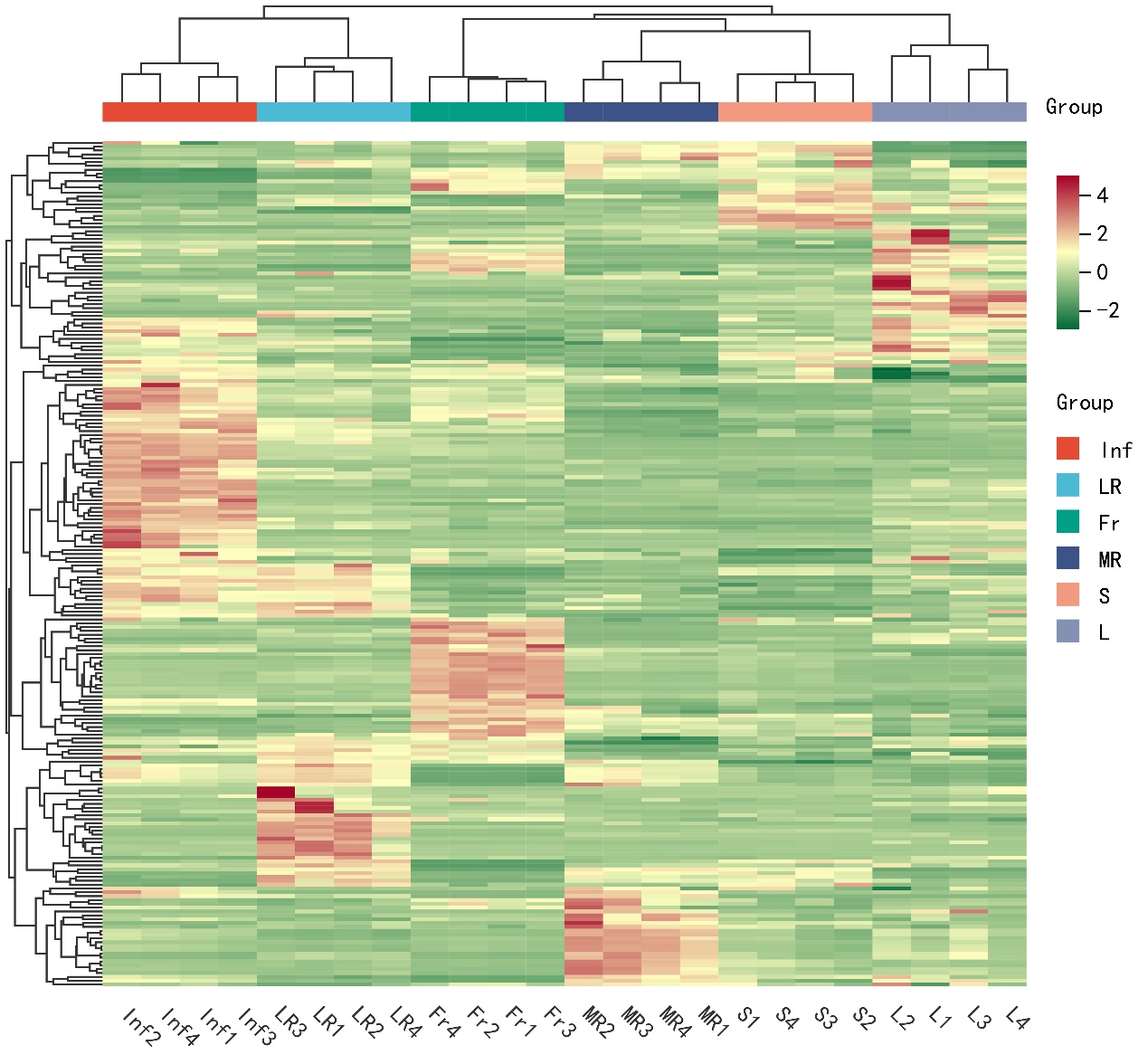


**Supplementary Figure 14.** Heatmap analysis of the levels of amino acids and derivatives.


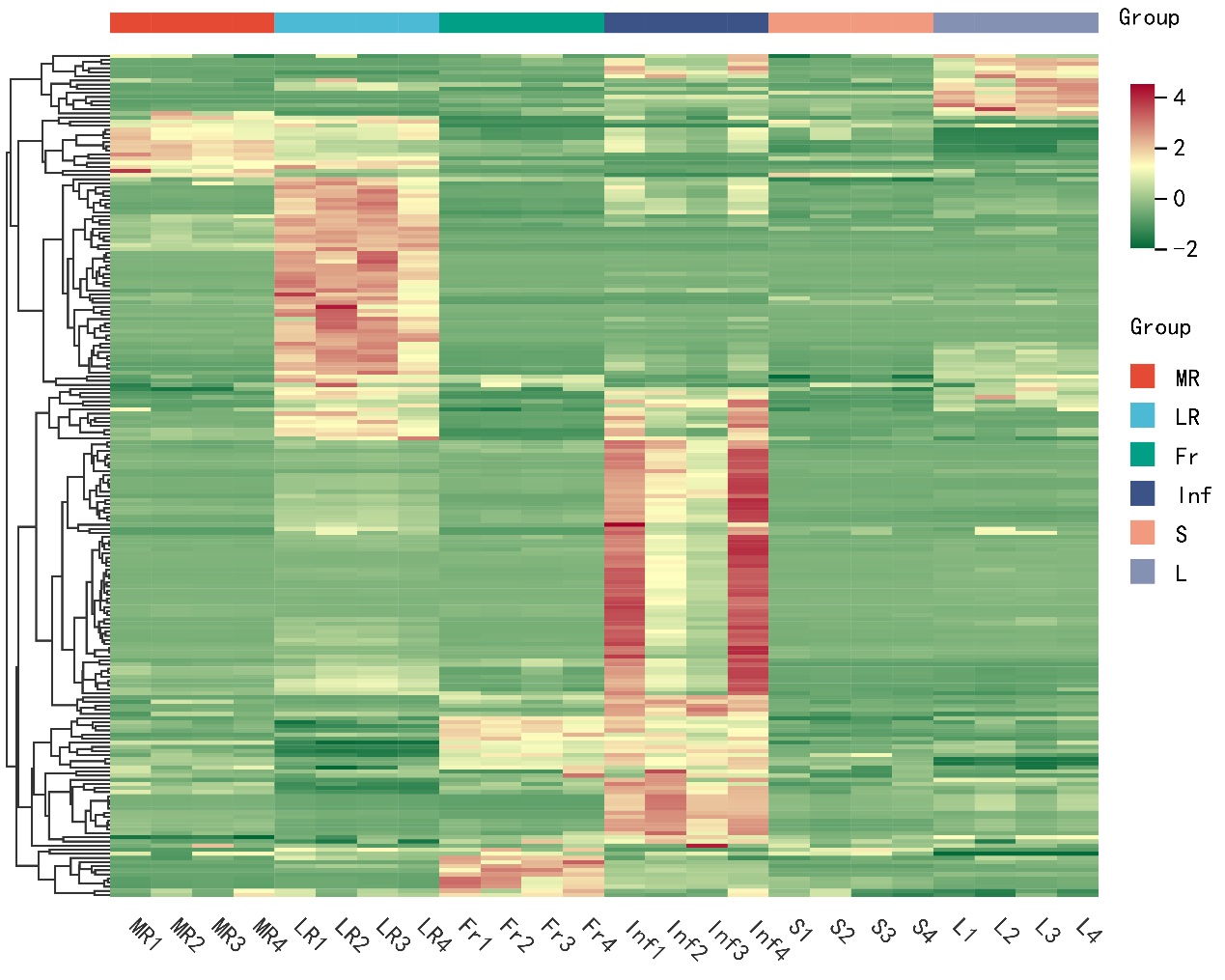


**Supplementary Figure 15.** Heatmap analysis of the levels of lipids.


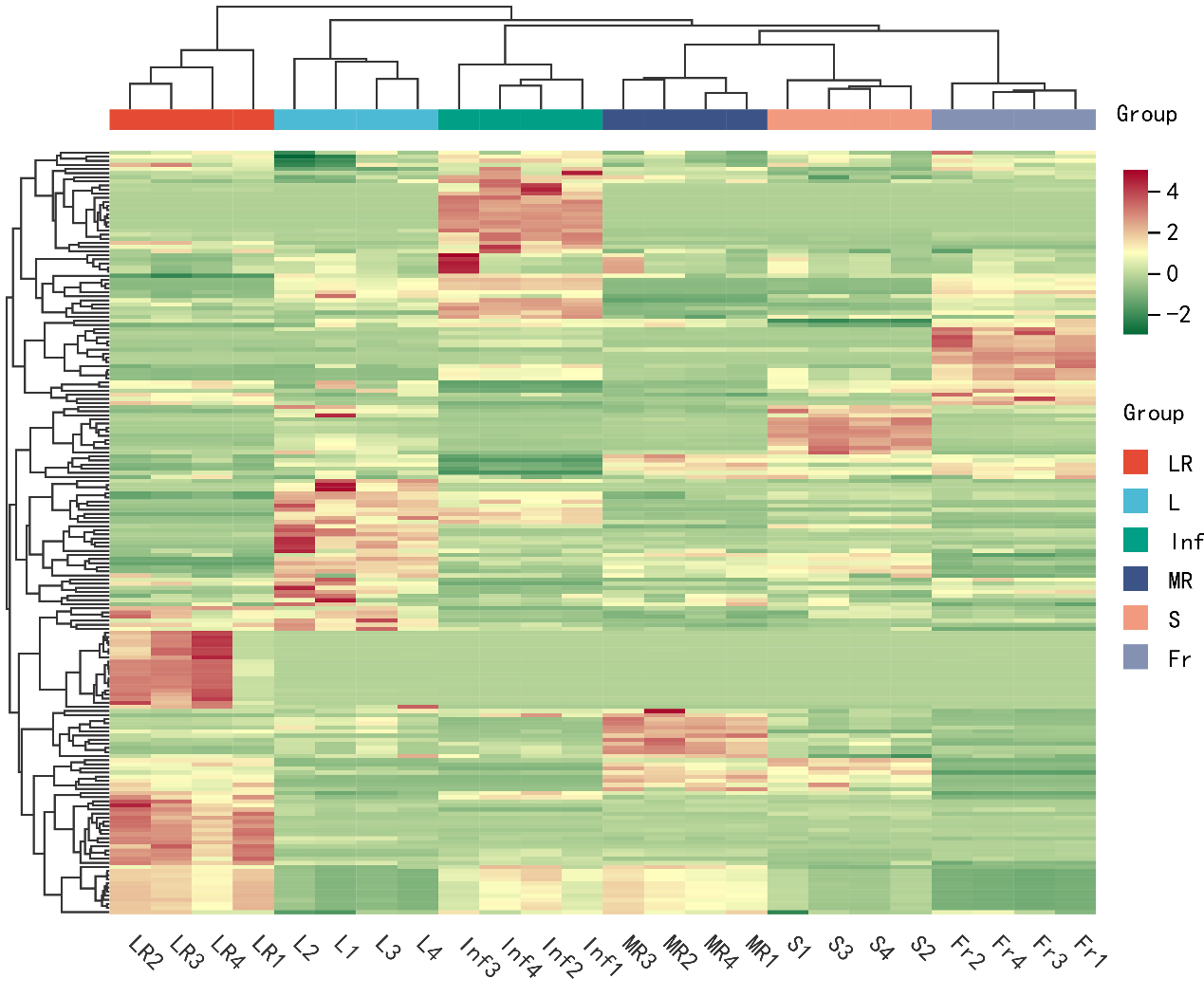


**Supplementary Figure 16.** Heatmap analysis of the levels of alkaloids.


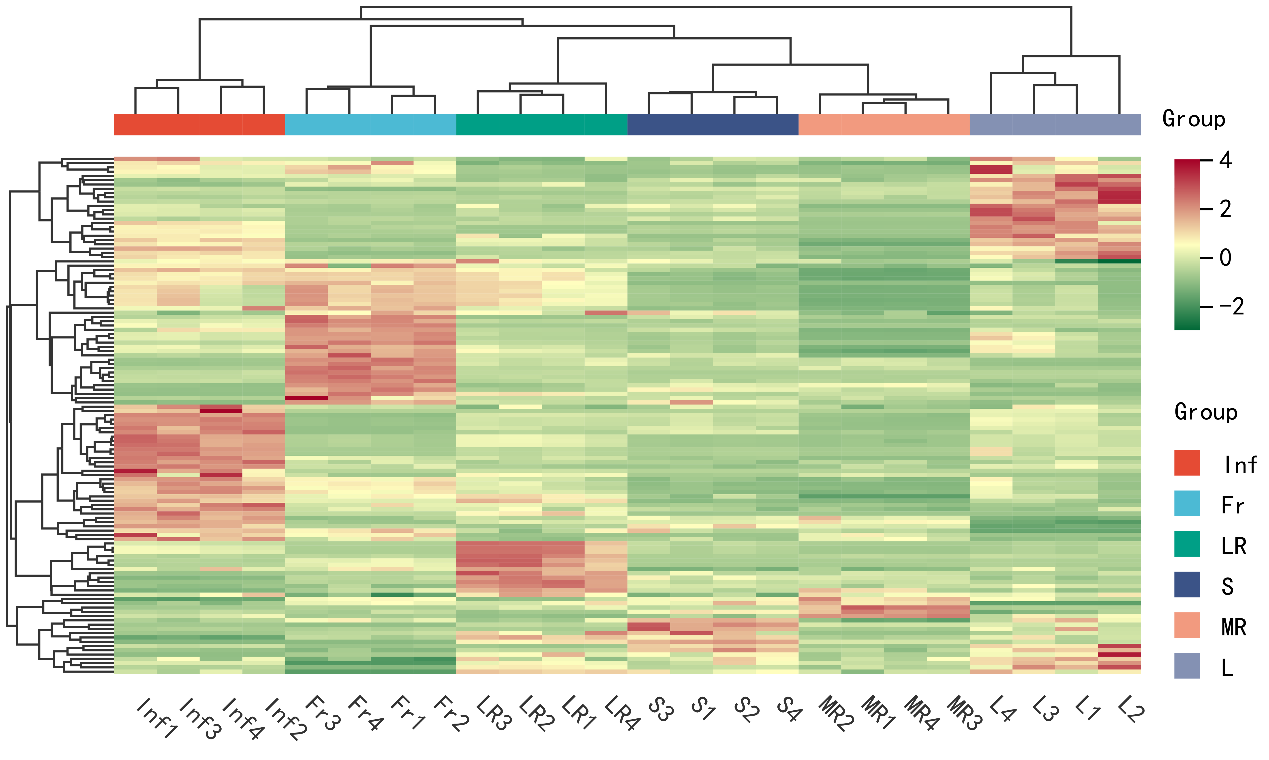


**Supplementary Figure 17.** Heatmap analysis of the levels of organic acids.


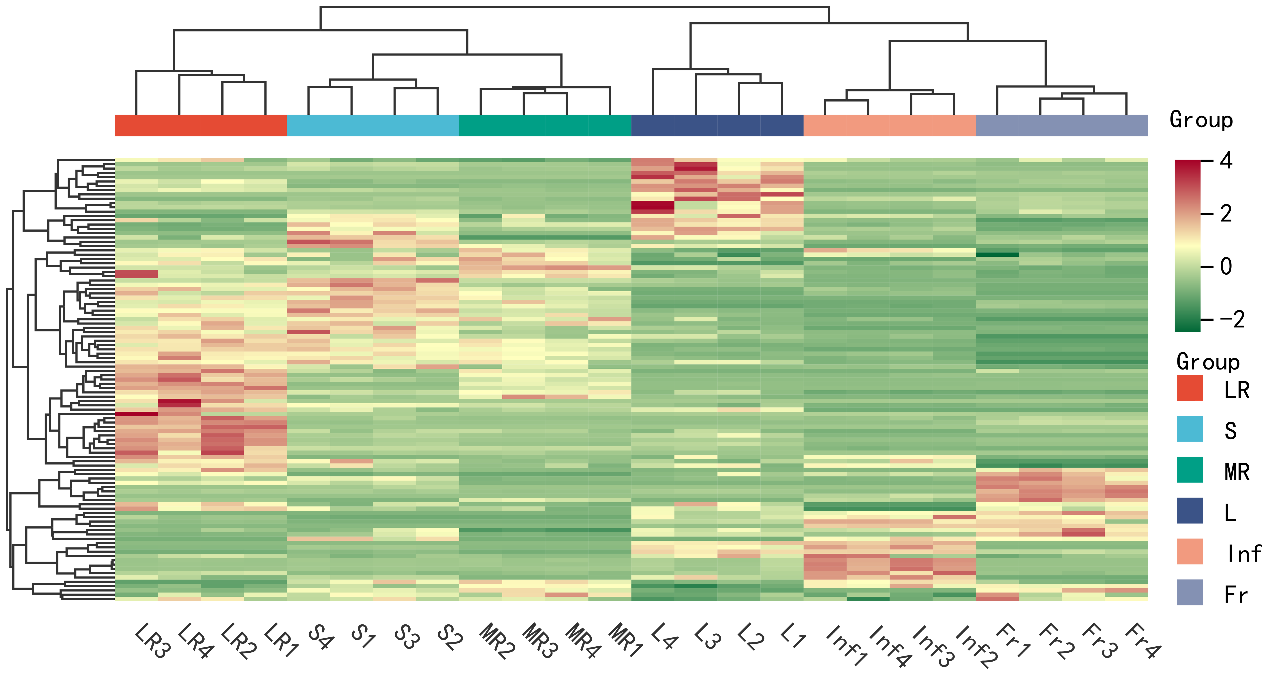
**Supplementary Figure 18.** Heatmap analysis of the levels of lignans and coumarins.


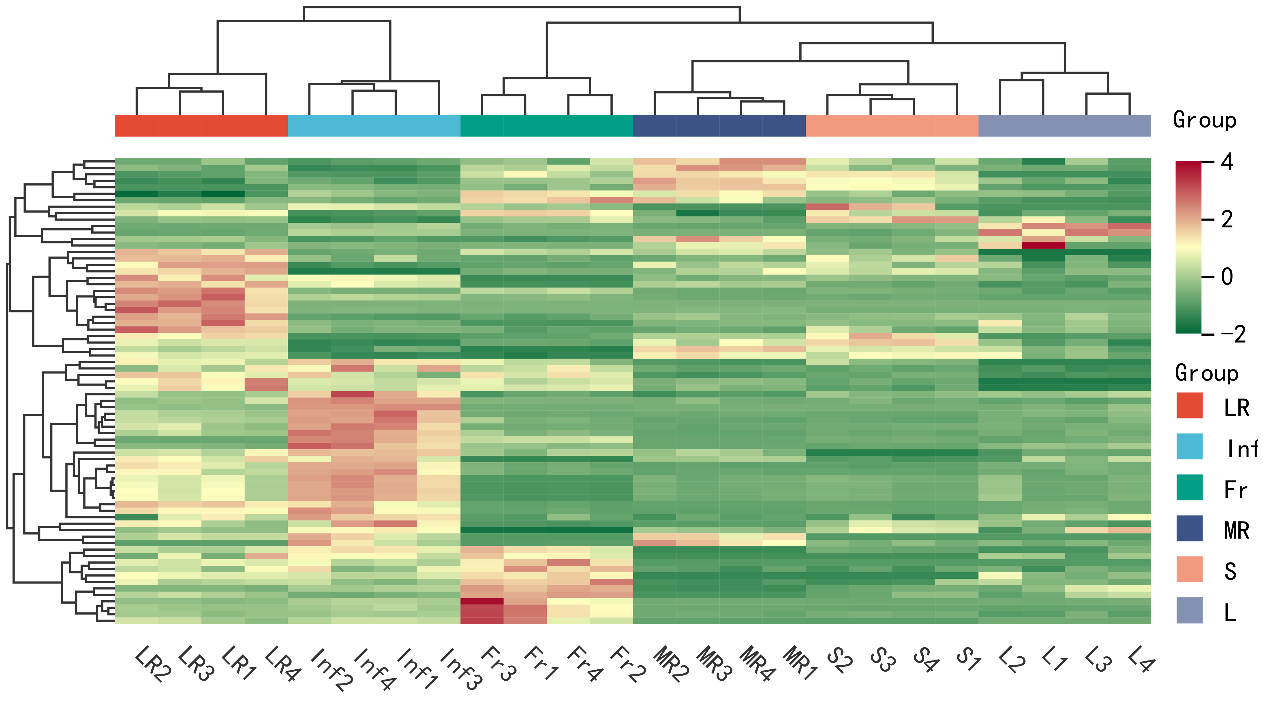


**Supplementary Figure 19.** Heatmap analysis of the levels of nucleotides and derivatives.


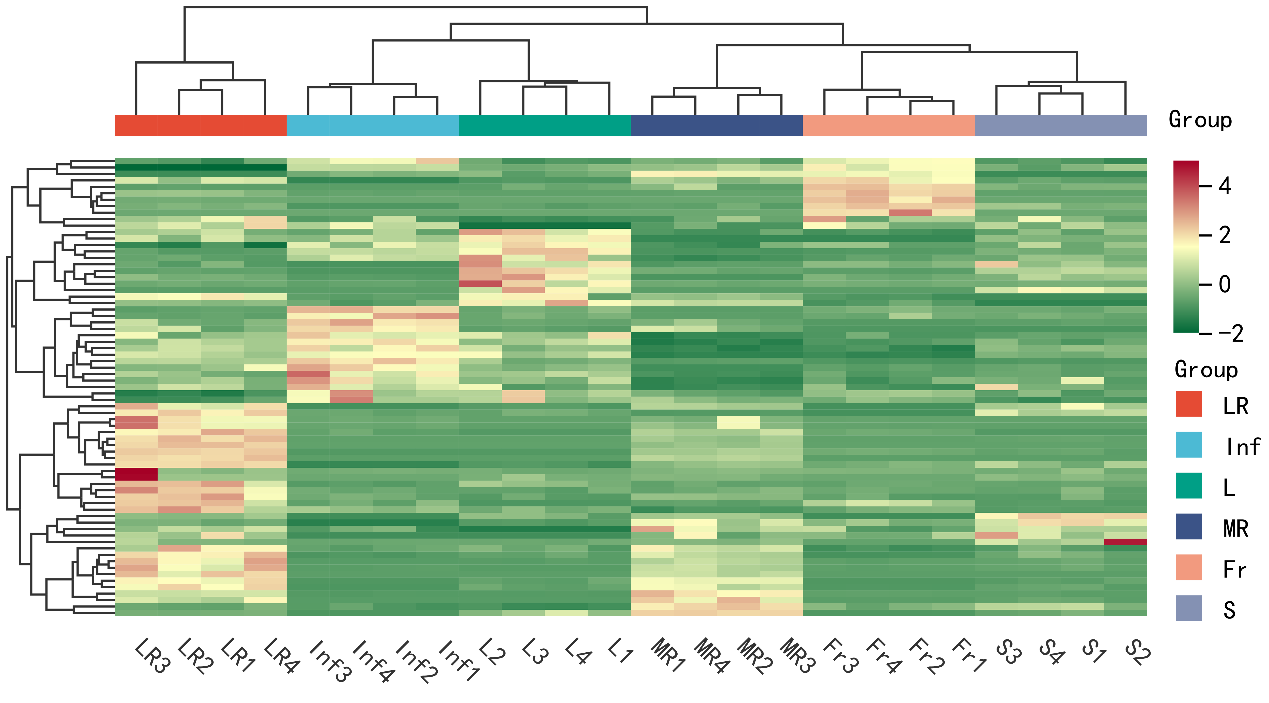


**Supplementary Figure 20.** Heatmap analysis of the levels of terpenoids.


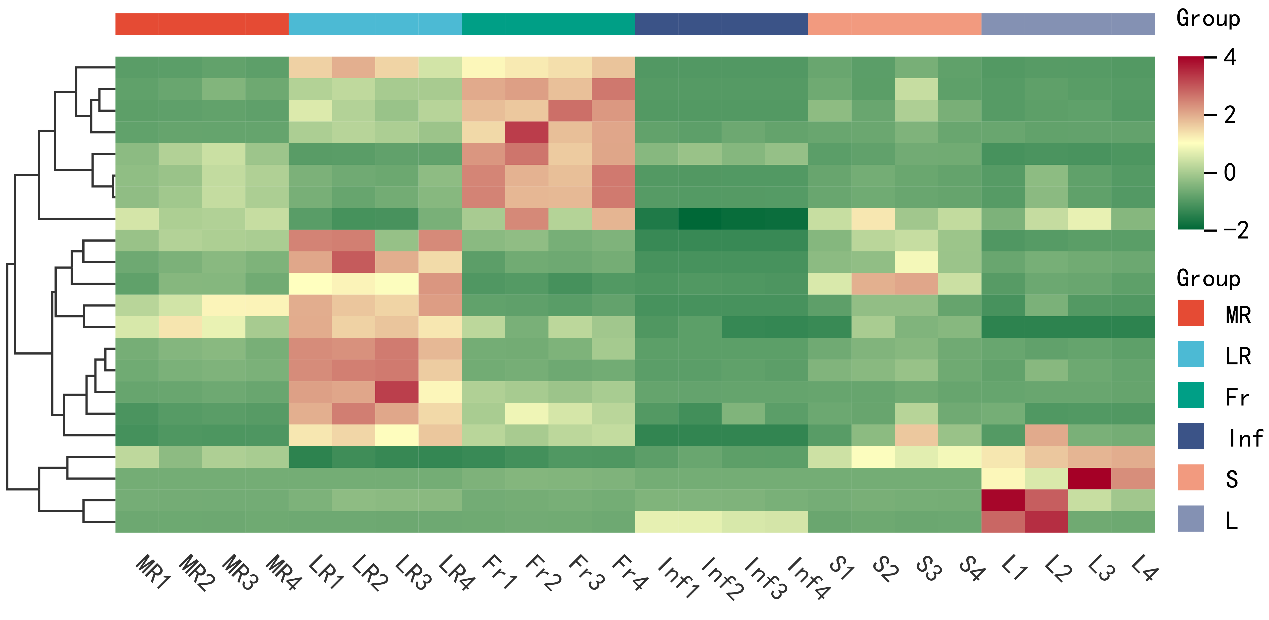


**Supplementary Figure 21.** Heatmap analysis of the levels of quinones.


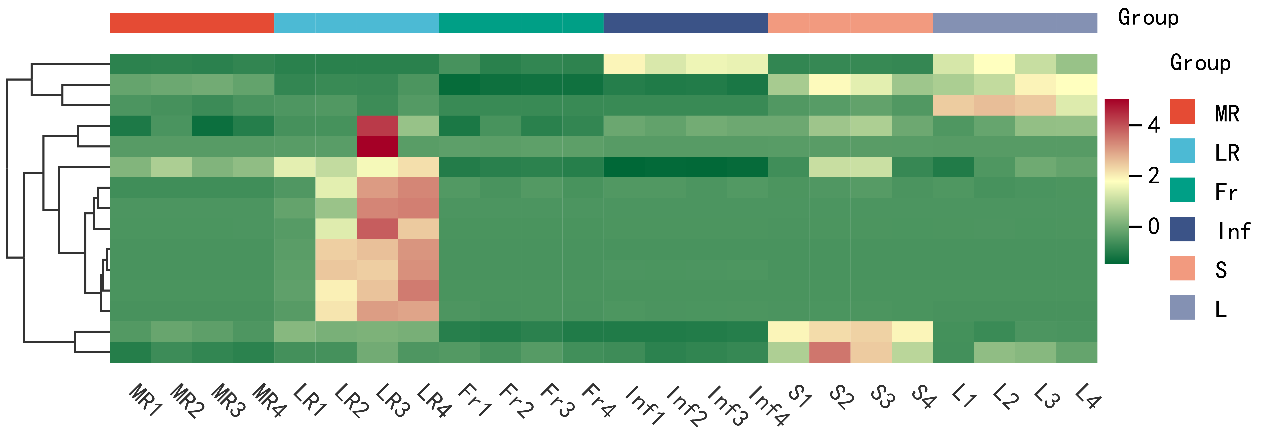


**Supplementary Figure 22.** Heatmap analysis of the levels of tannins.


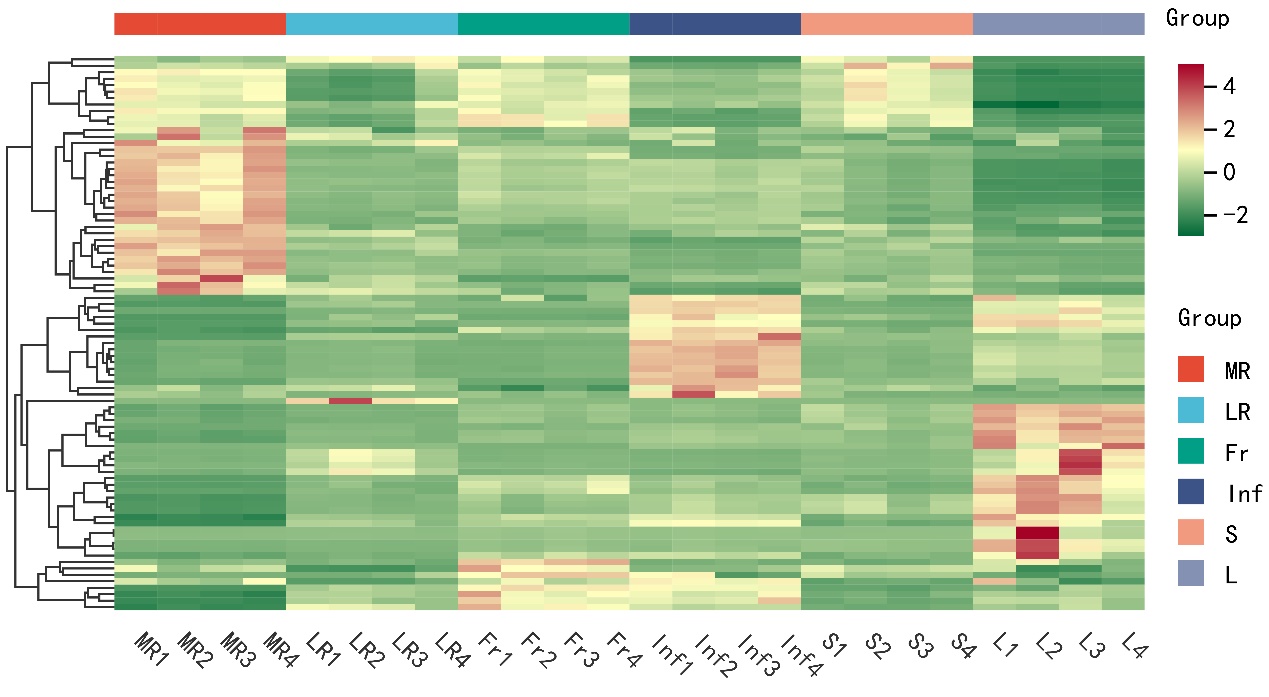


**Supplementary Figure 23.** Heatmap analysis of the levels of saccharides.


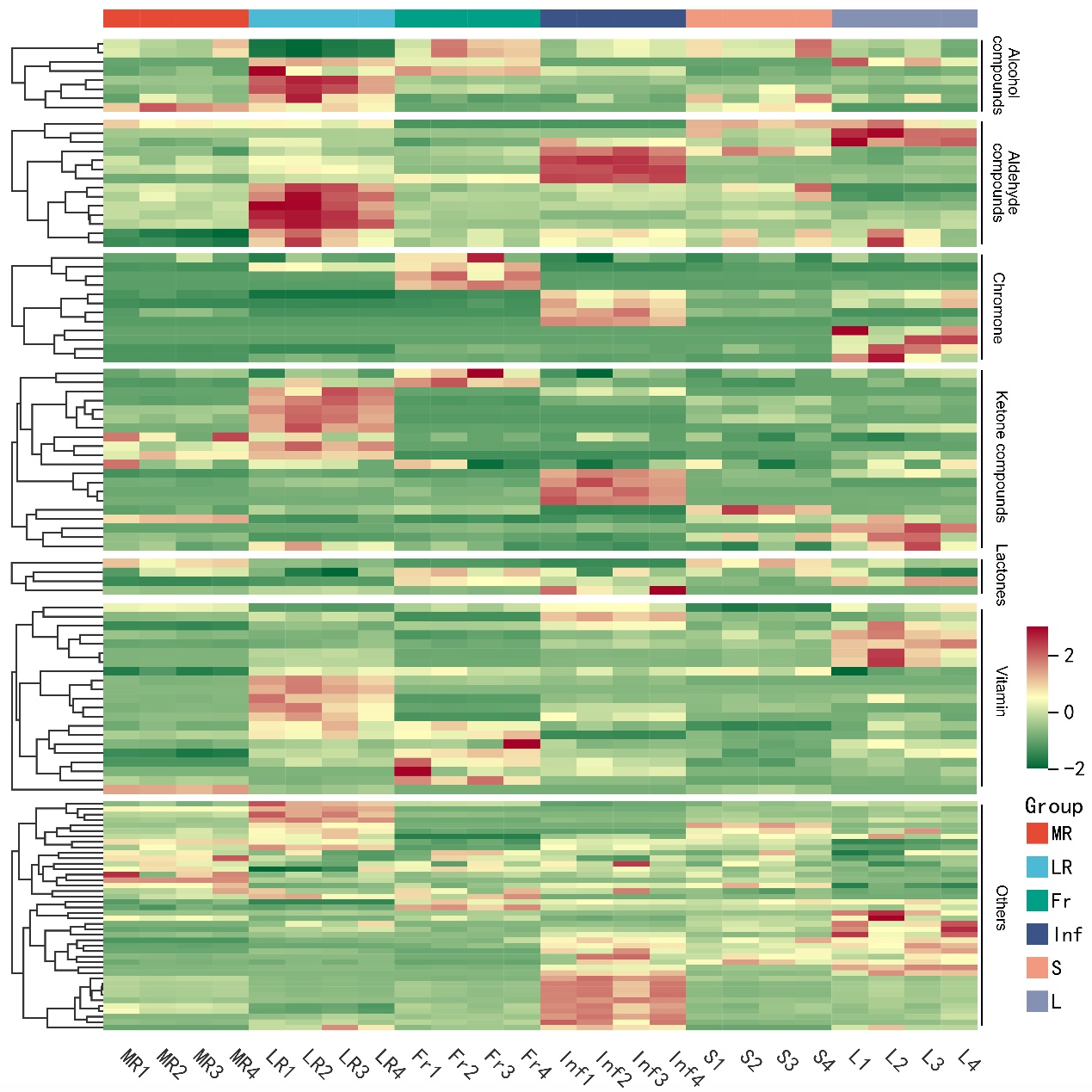


**Supplementary Figure 24.** Heatmap analysis of the levels of other categories of metabolites, including alcohol compounds, aldehyde compounds, chromone, ketone compounds, lactones, vitamin, and others.


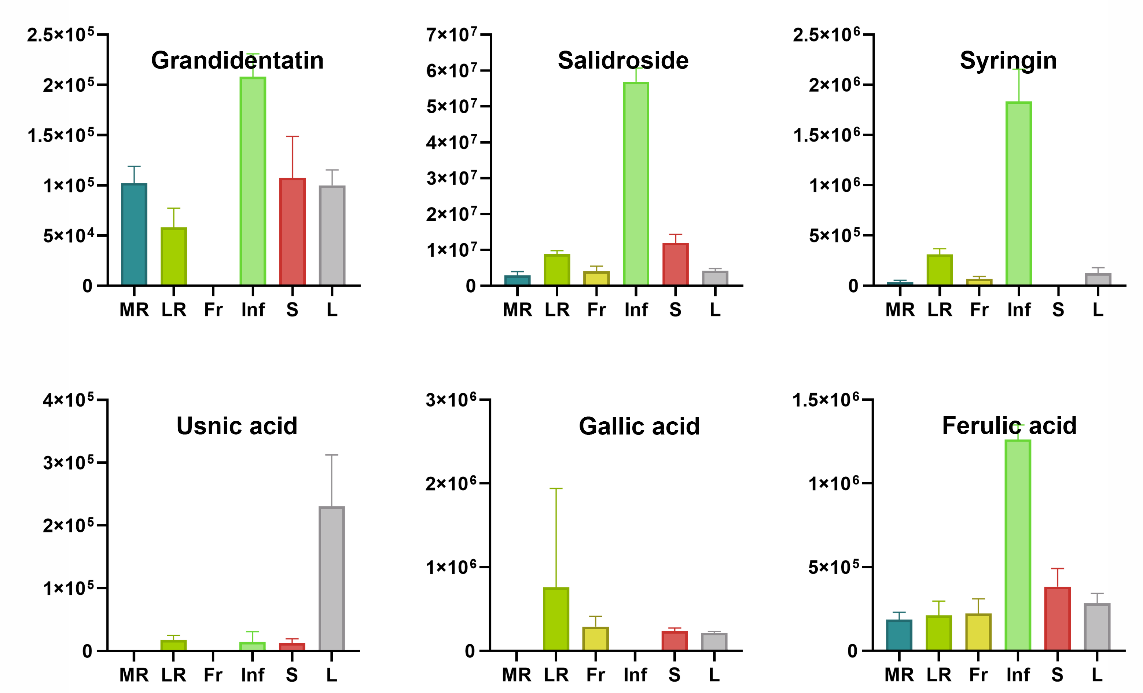


**Supplementary Figure 25.** The relative contents of the six health-promoting phenolic acids in different tissues of *Euchresta japonica*.


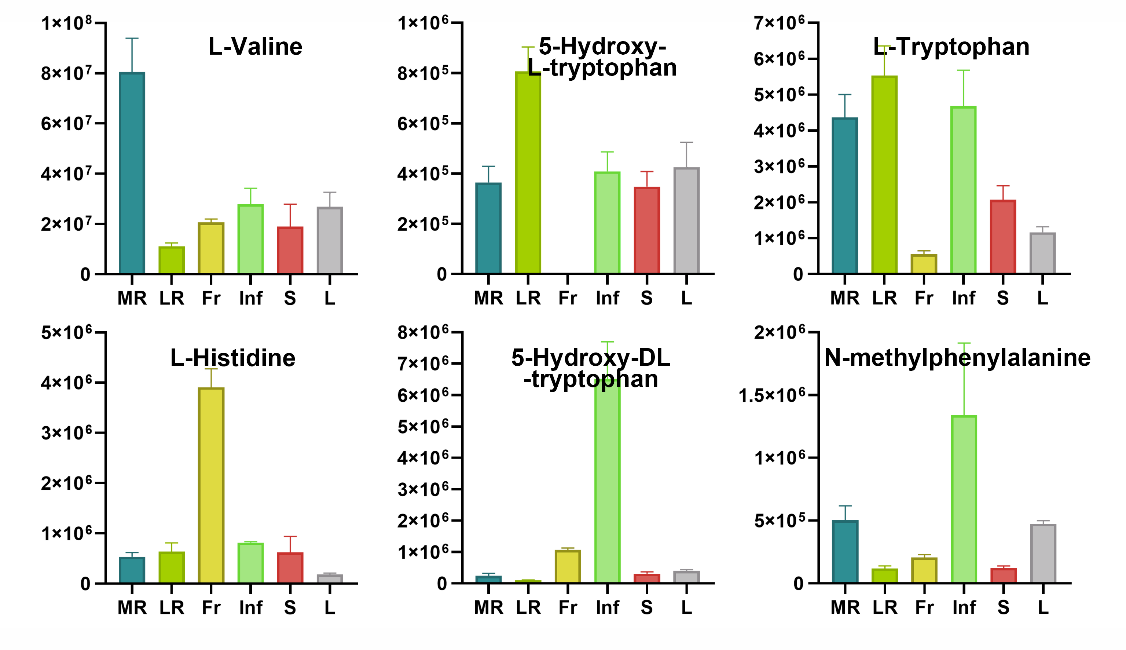


**Supplementary Figure 26.** The relative contents of the six health-promoting amino acids and derivatives in different tissues of *Euchresta japonica*.


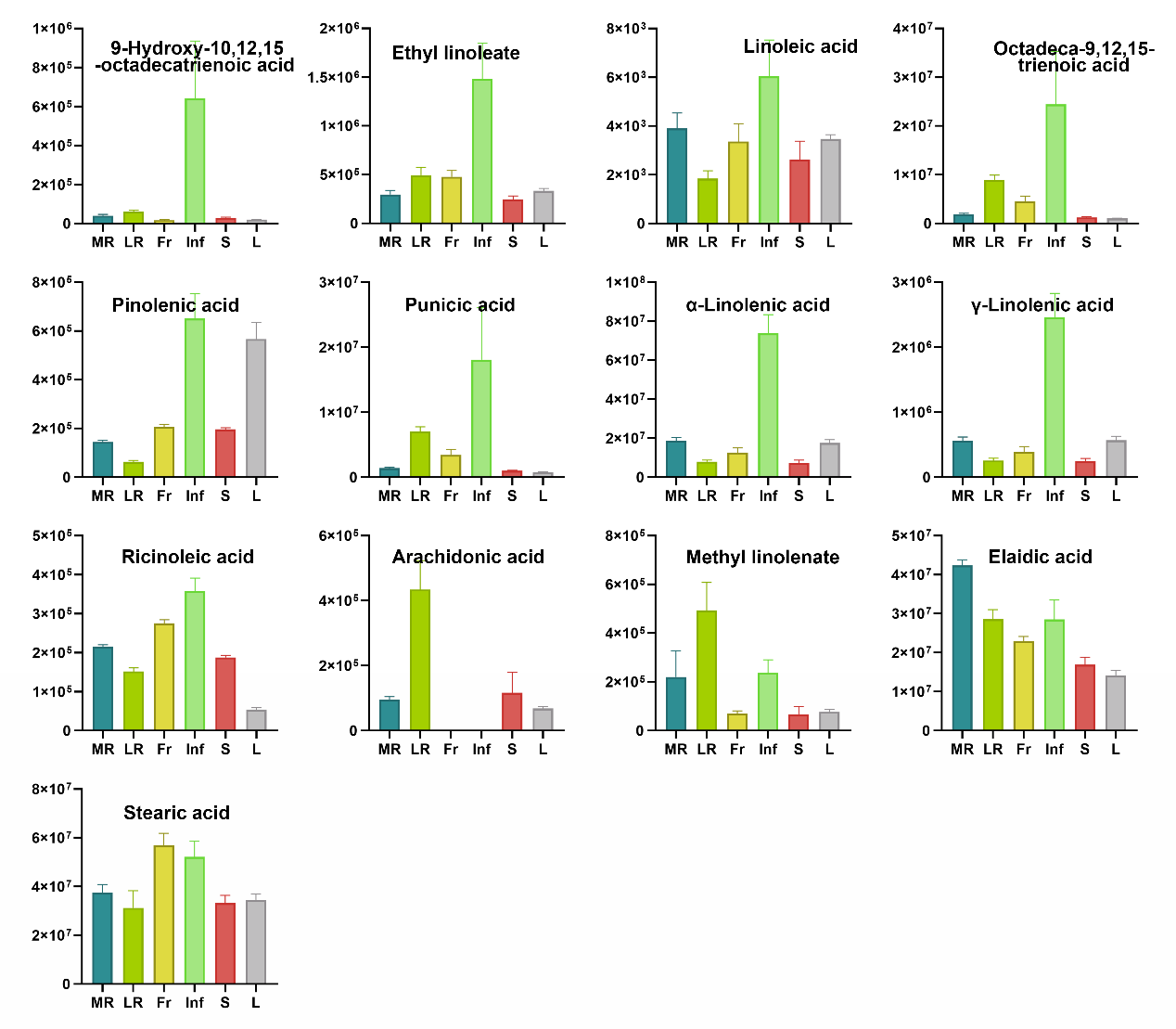


**Supplementary Figure 27.** The relative contents of the 13 health-promoting lipids in different tissues of *Euchresta japonica*.


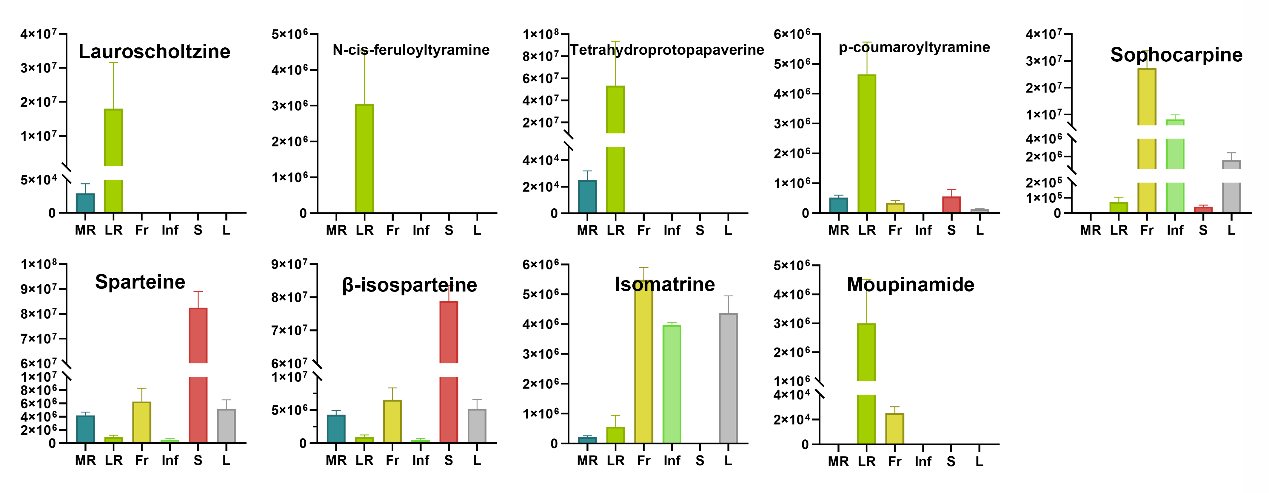


**Supplementary Figure 28.** The relative contents of the nine health-promoting alkaloids in different tissues of *Euchresta japonica*.


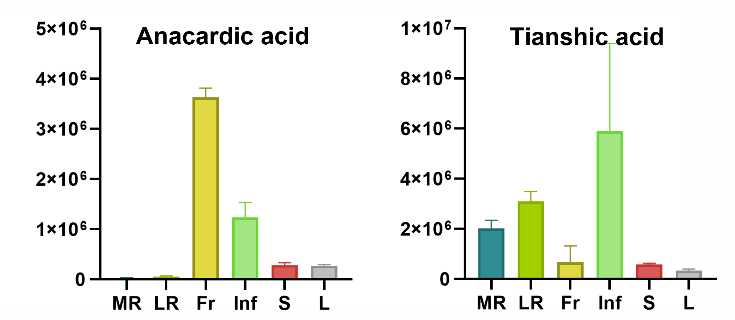


**Supplementary Figure 29.** The relative contents of the two health-promoting organic acids in different tissues of *Euchresta japonica*.


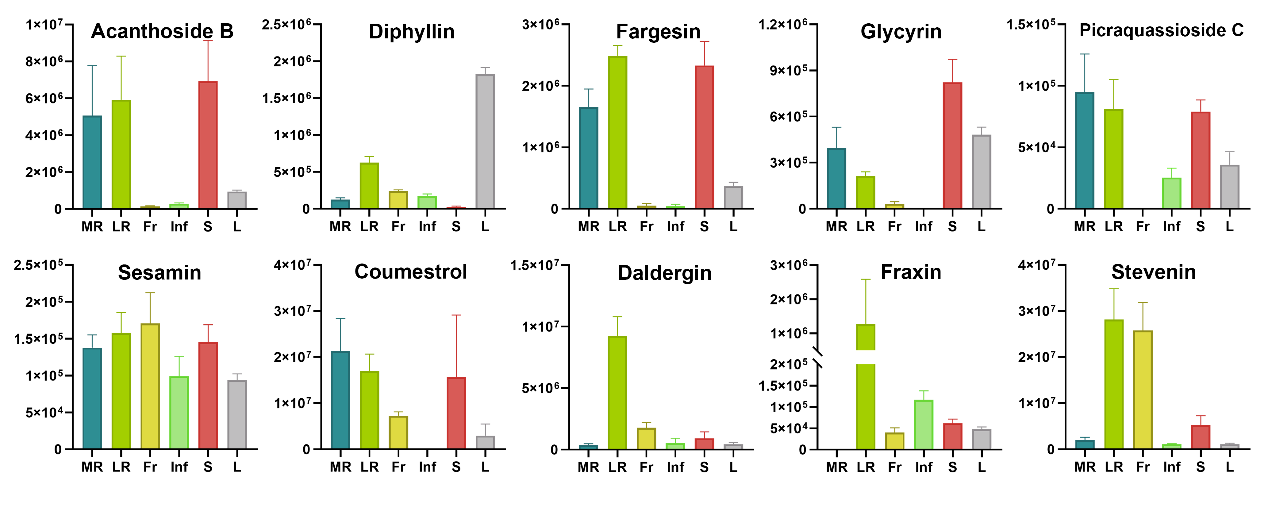


**Supplementary Figure 30.** The relative contents of the ten health-promoting lignans and coumarins in different tissues of *Euchresta japonica*.


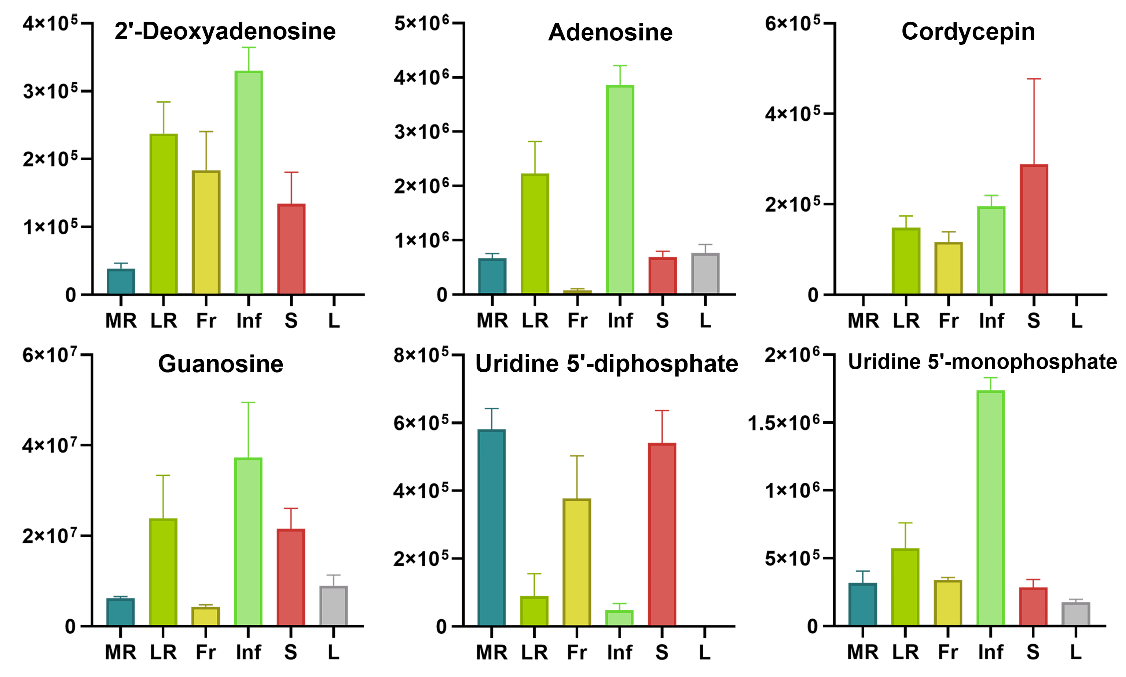


**Supplementary Figure 31.** The relative contents of the six health-promoting nucleotides and derivatives in different tissues of *Euchresta japonica*.


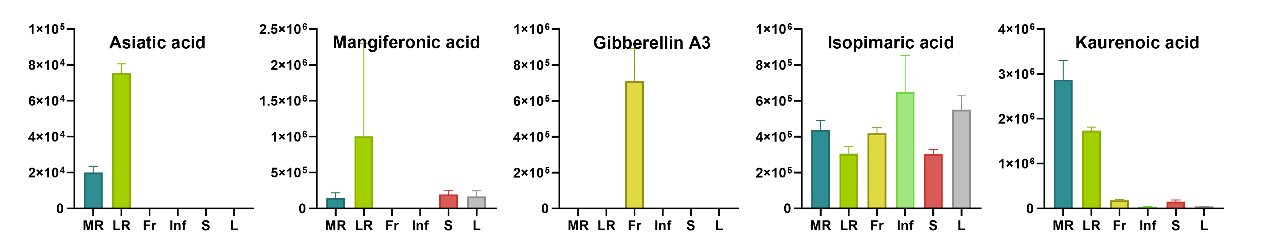


**Supplementary Figure 32.** The relative contents of the five health-promoting terpenoids in different tissues of *Euchresta japonica*.


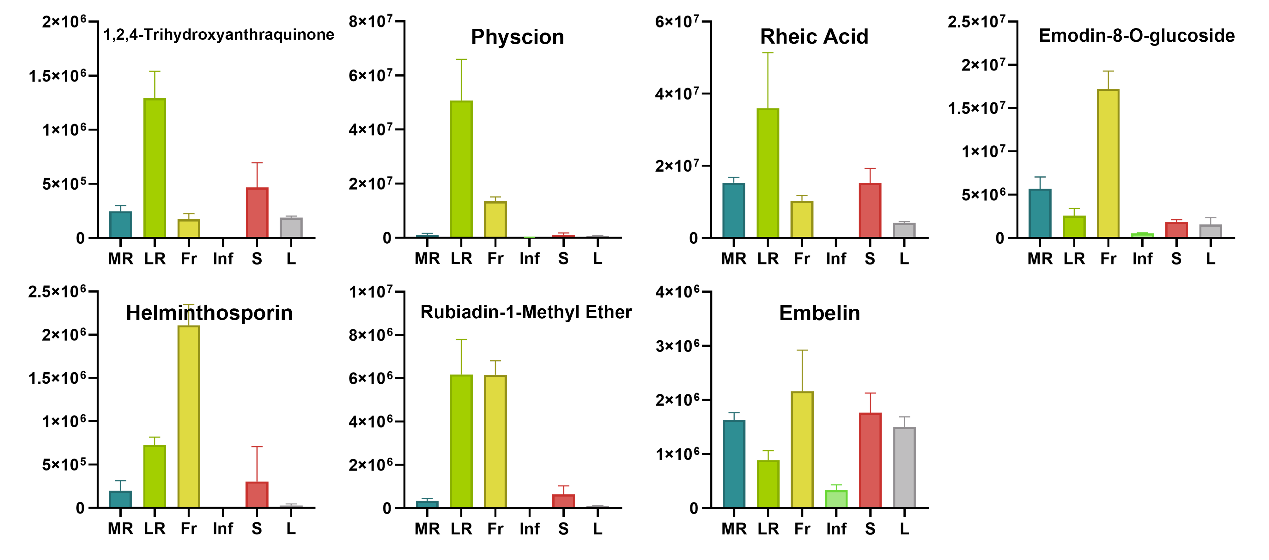
 **Supplementary Figure 33.** The relative contents of the seven health-promoting quinones in different tissues of *Euchresta japonica*.


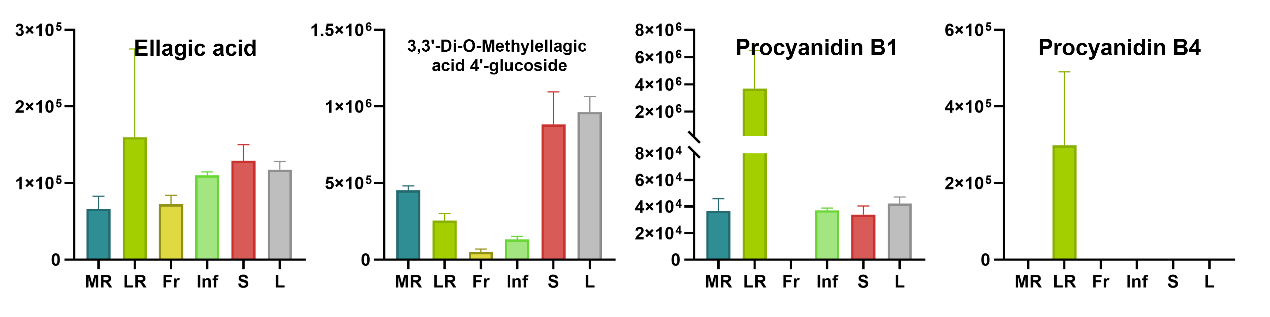


**Supplementary Figure 34.** The relative contents of the four health-promoting tannins in different tissues of *Euchresta japonica*.


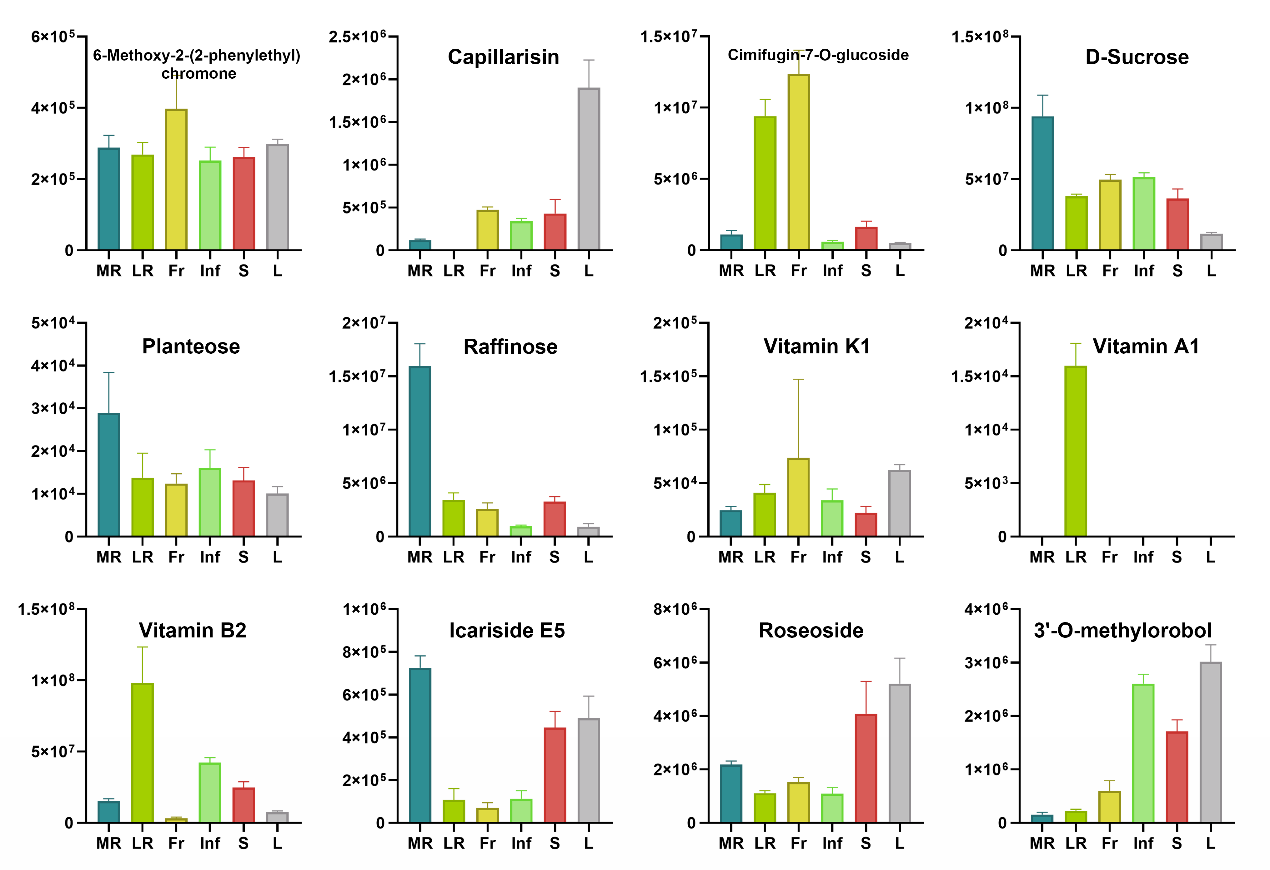


**Supplementary Figure 35.** The relative contents of the 12 health-promoting metabolites in different tissues of *Euchresta japonica*.


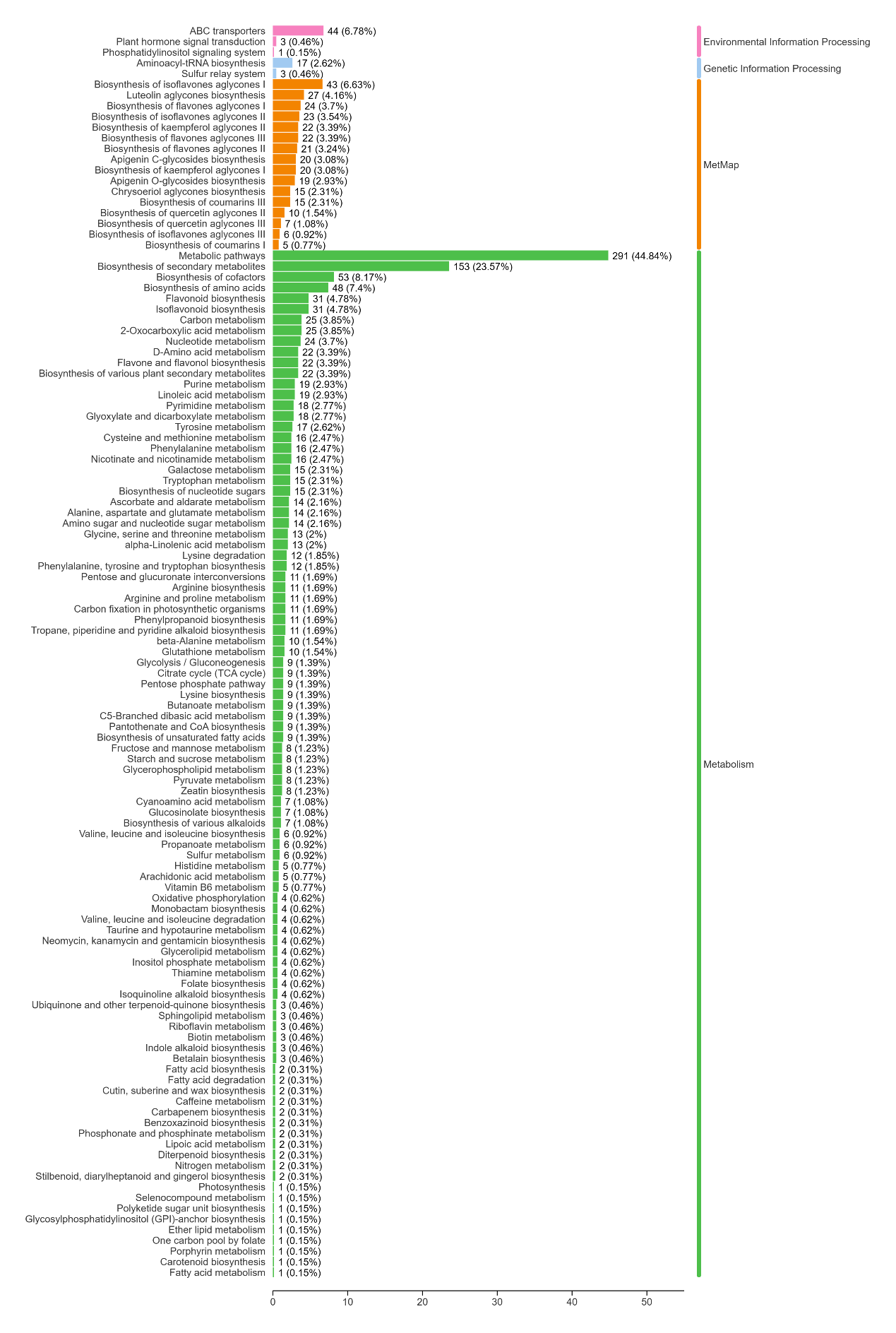


**Supplementary Figure 36.** KEGG classification of differentially expressed metabolites in *Euchresta japonica*.


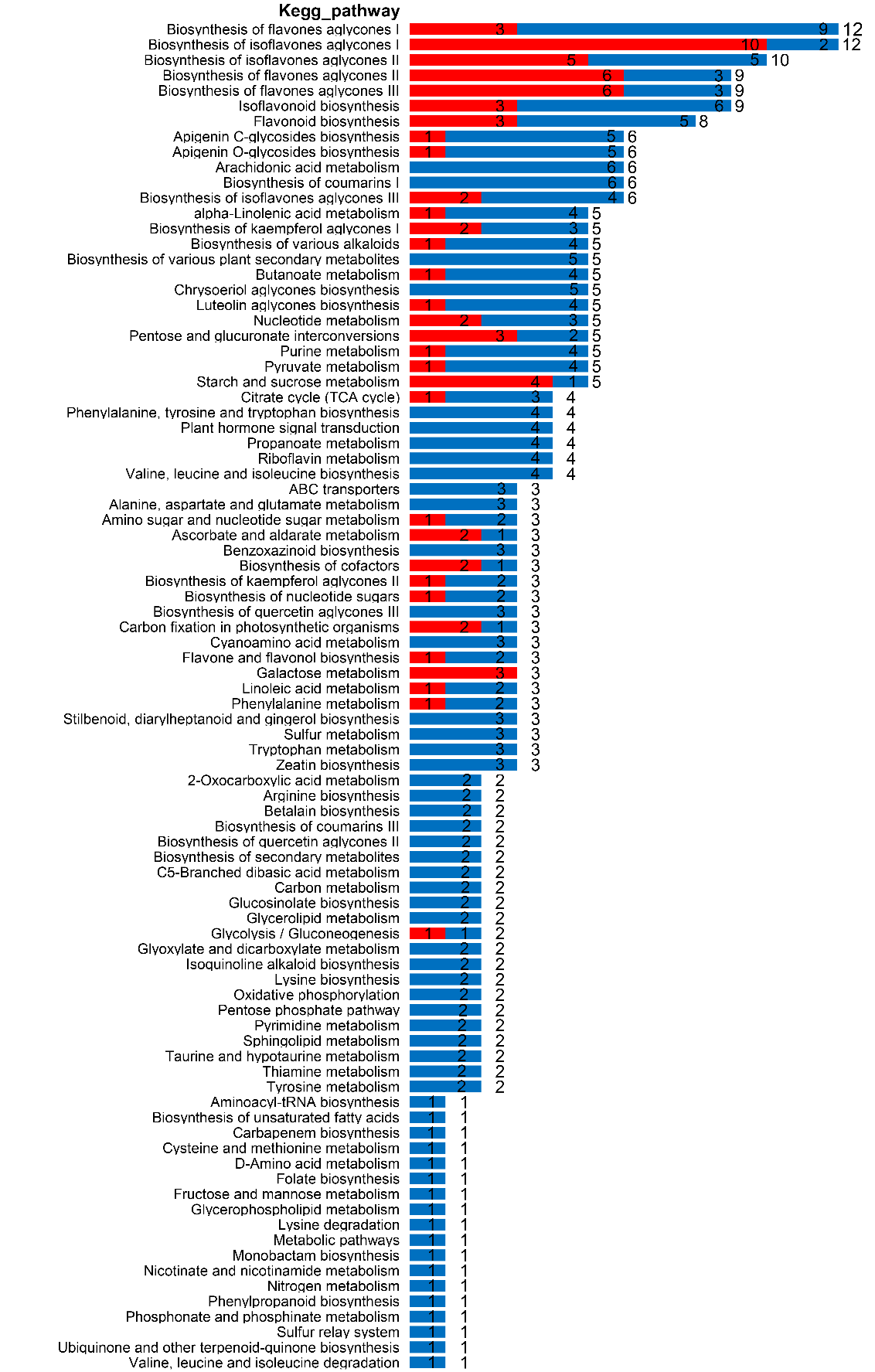


**Supplementary Figure 37.** Statistical analysis of the top 20 KEGG pathways in 15 pairwise comparison groups. The red bars represent the number of comparison groups in which the corresponding KEGG pathway is significantly enriched. The blue bars represent the number of comparison groups of the corresponding KEGG pathways that are not significantly enriched but are among the top 20 KEGG pathways.


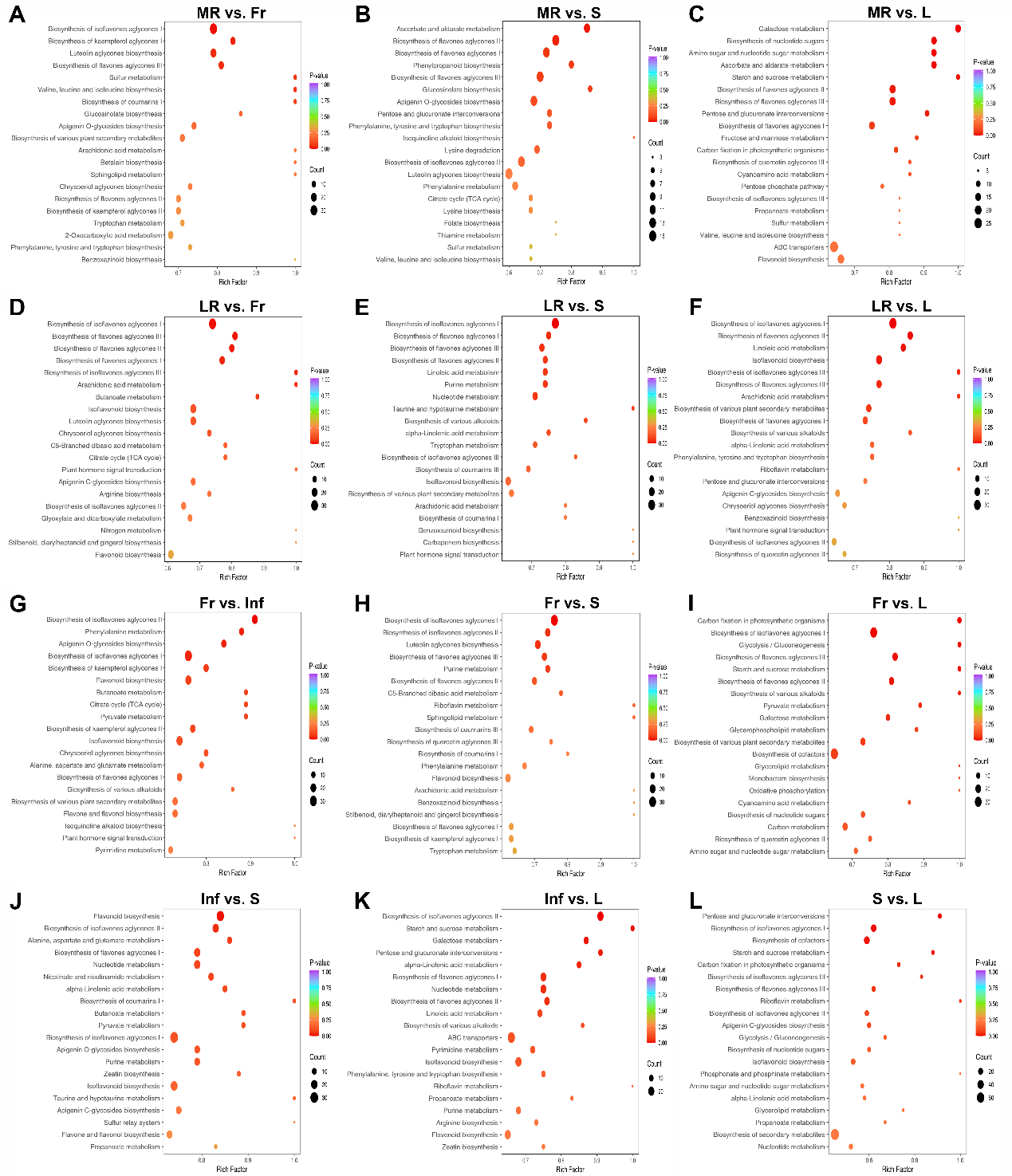


**Supplementary Figure 38.** KEGG annotations and enrichment of differentially expressed metabolites of each pairwise comparison of *Euchresta japonica*. (A-C) MR vs. Fr/S/L; (D-F), LR vs. Fr/S/L; (G-I), Fr vs. Inf/S/L; (J-K), Inf vs. S/L; (L), S vs. L.


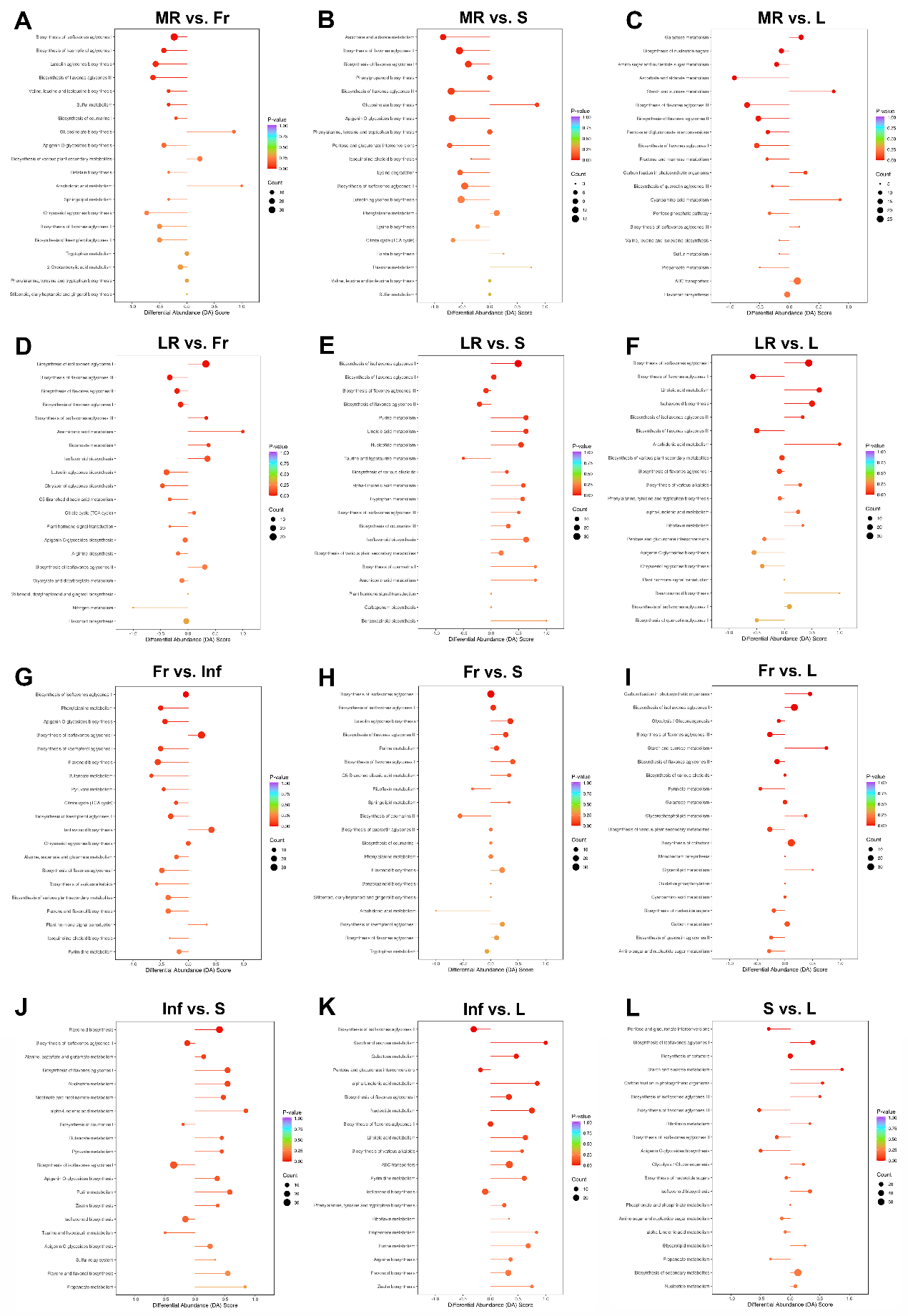


**Supplementary Figure 39.** Differential abundance (DA) score of differentially expressed metabolites of each pairwise comparison of *Euchresta japonica*. (A-C) MR vs. Fr/S/L; (D-F), LR vs. Fr/S/L; (G-I), Fr vs. Inf/S/L; (J-K), Inf vs. S/L; (L), S vs. L.
